# HAMC-ID: hybrid attention-based meta-classifier for intrusion detection

**DOI:** 10.1038/s41598-025-26631-8

**Published:** 2025-12-06

**Authors:** S. Antony Joseph Raj, M. Madiajagan

**Affiliations:** School of Computer Science and Engineering, Vellore Institute of Technology, Vellore, 632014 Tamil Nadu India

**Keywords:** Intrusion detection system (IDS), Attention mechanism, Meta-classifier, Bidirectional long Short-Term memory (BiLSTM), Extreme gradient boosting (XGBoost), Logistic regression (LR), Extra trees classifier (ET), Ensemble learning, Network security, Computational biology and bioinformatics, Mathematics and computing

## Abstract

Traditional IDS, which frequently lack flexibility and accuracy in diverse network scenarios, face significant difficulties from the growing complexity and frequency of cyber intimidations. To enhance detection performance, this study proposes a two-level stacking ensemble framework called HAMC-ID. At Level-0, three heterogeneous base classifiers—Extreme Gradient Boosting, Extra Trees, and Logistic Regression—are employed to capture diverse decision boundaries. At Level-1, a Bidirectional Long Short-Term Memory network with an integrated attention mechanism serves as the meta-classifier, dynamically aggregating meta-features such as logits, prediction confidence, and entropy to generate robust final predictions. The effectiveness of HAMC-ID is evaluated on two benchmark IDS datasets, UNSW-NB15 and CICIDS2017, for both binary and multiclass classification tasks. Experimental results demonstrate that HAMC-ID consistently outperforms individual classifiers and traditional ensemble approaches in terms of accuracy, precision, recall, and F1-score, thereby confirming its efficacy and versatility in practical cybersecurity applications.

## Introduction

Due to the quick development of the digital landscape and growing dependence on digital infrastructure, the frequency and sophistication of cyberattacks have dramatically increased. Antivirus software, firewalls, and access control rules are examples of traditional security methods that are becoming less effective against these changing threats. Because of this, intrusion detection systems, especially network-based IDS, are now vital parts of modern cybersecurity designs. To detect malicious behaviour, unauthorized access, and anomalies indicative of compromise, these systems endlessly analyse network traffic^1^. IDS solutions typically analyse network data to detect deviations from normal traffic behaviour. A prevailing method established by Machine learning to enhance IDS effectiveness^2^. Machine Learning-based models enable accurate classification of network traffic by learning complex patterns to differentiate between benign and intrusive behaviours. These models can be considered into single-classifier systems, which depend on on a single algorithm, and ensemble structures, which integrate many classifiers to exploit their individual strengths^3,4^.

Dietterich^5^ emphasized three foundational benefits of ensemble learning: (i) statistical, where ensemble models reduce variance and prevent overfitting; (ii) computational, where diverse learners can avoid convergence to poor local minima; and (iii) representational, where the ensemble captures a richer hypothesis space than a single model. These advantages have made ensemble methods increasingly popular in security applications.

Towards further improve classification reliability, attention mechanisms have been integrated into ensemble frameworks. In the context of meta-classifiers, attention dynamically reweights the outputs of base classifiers according to the input characteristics, thereby enabling context-aware decision-making^6^. This adaptive weighting is particularly critical in intrusion detection, where the diversity of traffic patterns across attack types and datasets challenges static fusion strategies.

In the direction of improve the correctness of detection and resilience, HAMC-ID propose a novel ensemble-based architecture that integrates attention-guided decision fusion. The significant contributions of proposed model are as follows:

- *Combining robust base classifiers* HAMC-ID combines XGBoost, Logistic Regression, and Extra Trees to create a robust ensemble that captures varied and complex attack behaviours.
- *Dynamic attention-based fusion* An embedded attention mechanism with meta-classifier dynamically assigns context-aware weights to base classifier outputs, optimizing predictions across different traffic profiles.
- *Enhanced performance in imbalanced settings* Specifically in multiclass intrusion detection scenarios, the proposed model shows extraordinary ability in identifying complex and minority-class intimidations.

## Related works

Examine efforts in the intrusion detection segment have increased dramatically in an effort to create more resilient and flexible defences against the growing threat of cyberattacks. From federated learning and attention mechanisms to deep learning and ensemble techniques, researchers have investigated a variety of approaches that have advanced IDS capabilities while frequently encountering particular difficulties with computational efficiency, scalability, and adaptability to changing attack landscapes. Setting the scene for the innovative methodology suggested in this study, this section offers a thorough summary of recent contributions, stressing both their advantages and disadvantages.

To improve intrusion detection, academics have so far tried a variety of approaches, from complex ensemble techniques and distributed learning paradigms to deep learning architectures. The goal of increasing accuracy, lowering false positives, and guaranteeing real-time applicability in progressively complex network environments is a common theme among many of these initiatives. The prominent academic databases and conference proceedings from recent years with keywords like “intrusion detection systems,” “ensemble learning for IDS,” “deep learning for cybersecurity,” “attention mechanisms in IDS,” “federated learning for intrusion detection,” and “meta-learning for network security” in order to conduct our literature survey. The results were presented chronologically to show the evolution of research.

A high precision ensemble deep learning recommended by Al-Abassi et al.^7^ to detect cyberattacks and attained an extraordinary detection rate of more than 96%, in Industrial Control Systems. Yet, due to with inadequate resources and high computational cost, so it detects inappropriately, and its processing intensity causes scaling issues. Chatterjee and Hanawal^8^ presented a federated hybrid ensemble intrusion detection system, utilizing a federated learning-based hybrid ensemble classifier (PHEC) designed for IoT security environments to enhance detection across several network nodes. While this method aids scalability, it struggles to adapt to newly changing attack patterns. Sunil Raj et al.^9^ introduced RTPIDS, a real-time adaptive intrusion detection system for IoT that integrates federated learning and blockchain to ensure decentralized training and trust management. While this approach enhances privacy and collaboration, it also increases communication and computational overhead.

A Snapshot Ensemble Learning model with group convolution was presented with two datasets namely first NSL-KDD, second UNSW-NB15 by Wang et al.^10^ showed excellent accuracy (around 85%). Still, the idea reliance on static snapshots limits its ability to respond to new attack types, which reduces its applicability in dynamic scenarios. ARLIF-IDS, created by Fitni et al.^11^, combines isolation forests with attention processes to enhance anomaly detection. Although it is quite effective at identifying new assaults, its use is limited when labeled data is available because it is mostly intended for unsupervised anomaly detection. Lin et al.^12^ demonstrated an ensemble architecture for intrusion detection based on hypergraphs, achieving up to 99% detection accuracy using the CICIDS2017 dataset. However, its lack of dynamic ensemble optimization prevents real-time adaptability to changing traffic patterns. A stacking ensemble model that incorporates classifiers such as GNB and LR was proposed by Thockchom et al.^3^. Even though it performed satisfactorily on CICIDS2017, its static classifier fusion methodology limits its ability to react to quickly evolving attack patterns.

Few-shot IDS proposed by Sun et al.^13^ constructed on Prototypical Capsule Networks that strong alone on two datasets specifically NSL-KDD then CICIDS2017 nevertheless struggled on diversity in addition generalization across attack types.CNN-GWO-Voting hybrid by Ji et al.^6^, which combines feature extraction with CNN by using Grey Wolf Optimizer to select features. Despite the model use on CICIDS2017 dataset shows strong performance, its reliance on static feature selection and classifier fusion techniques limits its real-time flexibility. A deep learning (DL)-based IDS proposed by Vinayakumar et al.^14^ which integrates feedforward deep neural networks (DNNs) and achieved high detection rates across benchmark datasets including UNSW-NB15. However, it lacks sequential modeling capabilities like BiLSTM, limiting its adaptability to temporal dependencies in traffic flows.

An IDS model was introduced by Jouhari et al.^15^ with CNN-BiLSTM tailored for resource-constrained Internet of Things systems. Their approach significantly improved temporal pattern recognition, yet the model does not incorporate attention-based fusion, limiting its flexibility in adaptively weighing diverse classifier outputs. A dynamic attention-based fusion model was introduced by He et al.^16^, which utilized generation-enhanced learning for few-shot intrusion detection. While effective for low-data regimes, the absence of a stacking ensemble and rich meta-feature integration reduces its performance across full-spectrum IDS scenarios.

An ensemble of autoencoders by Mirsky et al.^17^ proposed Kitsune, for lightweight, real-time IDS. Even though this model works effectively for basic attacks, it has trouble identifying more intricate, multi-phase cyberattacks. Recurrent neural networks (RNNs) with attention mechanisms were investigated by Brown et al.^18^ for log anomaly detection; however, their scalability and capacity to manage dynamic attack types are restricted. Osanaiye et al. developed a method for ensemble-based multi-filter towards select feature^19^ for cloud-based Distributed Denial of Service detection. Although it increased the accuracy of DDoS detection, it did not address the unpredictability of other attacks or provide real-time flexibility in response to changing attack tactics.

For intrusion detection, Wang et al.^20^ used triplet graph convolutional networks (GCN) in conjunction with few-shot learning. The model struggled with novel assault patterns or a variety of traffic situations, although it did well in low-sample detection tasks. First, a lightweight attention-based IDS technique was contributed by Laghrissi et al.^21^ However, its fixed attention mechanism restricts its capacity to adapt to intricate and multi-stage attacks.

A fresh dataset was used get high detection rates were attained by Mahfouz et al.^22^ when they implemented intrusion detection using ensemble classifiers. However, proposed model is not real-time adaptive and has trouble scaling to large networks.

To create an IDS for IoT contexts, Alghamdi et al.^23^ integrated deep learning with lambda architecture. Although their model demonstrated scalability, stale data and its incapacity to adapt dynamically to changing attack types caused performance decrease. A few-shot IDS proposed by Iliyasu et al.^20^ based on supervised autoencoders. Proposed model performed well with limited data, but it had trouble with diverse attack characteristics and lacked real-time adaptability. For fog computing an ensemble DL model was proposed by Kalaivani et al.^21^. It showed potential in decentralized settings but struggled to identify changing attack types and lacked adaptability for dynamic situations.

In order to improve feature relevance for methods for intrusion detection, Bhargava and Akuthota^24^ integrated deep learning with ensemble feature selection; nonetheless, they were unable to adapt dynamically to changing attack patterns. A thorough study comparing individual machine learning models and ensemble techniques by Bibers et al. conducted research for network intrusion detection^23^, emphasizing the necessity of dynamically adjusting ensemble structures to evolving attack patterns. For 5G-enabled industrial networks, A meta-learning-based few-shot IDS algorithm presented by Yan et al.^25^ to improve identification of novel attacks but struggled to generalize to different traffic patterns. A Transformer-based model introduced for cloud security IDS by Long et al.^26^. It performed well but had a high computational cost, which limited its scalability for real-time detection.

Recent gradient boosting approaches have also been explored. Wang et al.^27^ proposed a LightGBM-based two-stage pipeline (STG2P) combining an improved LightGBM classifier with K-means clustering to enhance detection accuracy and reduce false positives. While achieving high detection rates, this model still struggles with imbalanced traffic and lacks temporal modeling capabilities, limiting its adaptability in dynamic network environments.

The suggested method introduces a lightweight attention-based fusion that improves real-time detection performance, decreases computational overhead, and increases scalability in contrast to the deep multi-architectural approach^28^, which depends on heavy stacked models, and ASRL^29^, which stresses reinforcement-driven optimization. To help illustrate how the research was carried out, Table 1 presents the data in chronological order and highlights some of the important linked articles that have influenced this paper. The pieces have evolved throughout time.

**Table 1:** Summary of existing IDS models and limitations.

Author	Model	Limitations
Abassi et al.^7^	Ensemble Deep Learning for ICS	High false positives and lack of strong ensemble techniques in industrial systems.
Chatterjee et al.^8^	Federated Hybrid Ensemble IDS	Lacks adaptability and ensemble diversity in centralized models.
Wang et al.^10^	Group Convolution + Snapshot Ensemble	Static ensemble without contextual adaptability.
Fitni et al.^11^	ARLIF-IDS (Attention-Augmented Isolation Forest)	Weak attention modeling; disregards feature importance in ensemble.
Lin et al.^12^	Hypergraph ML Ensemble IDS	Static models fail against dynamic attack behaviors
Thockchom et al.^3^	Voting-Based Dynamic Ensemble	Lacks learning-based adaptability in static voting systems
Sun et al.^13^	Few-Shot Capsule Network with Attention	Generalization is poor with small labeled datasets
Ji et al.^6^	CNN-GWO-Voting Hybrid IDS	No context-aware meta-layer; rigid across datasets.
Vinayakumar et al.^14^	Deep Learning IDS (DNN)	Performs well on benchmarks but lacks temporal modeling.
Jouhari et al.^15^	CNN-BiLSTM IDS for IoT	Enhances temporal detection but lacks dynamic attention-based fusion.
He et al.^16^	Generation-Enhanced Attention Fusion	Dynamic fusion for few-shot tasks; lacks full-stack ensemble learning
Wang et al.^27^	LightGBM-based Two-Stage Pipeline (STG2P)	High detection rates, but struggles with imbalanced data and lacks temporal modeling; limited adaptability in dynamic network environments.

The majority of current IDS models have one or more significant drawbacks, as shown by the analysis in Table 1: (i) static ensemble learning without contextual adaptability^3,10,12^, (ii) weak temporal or attention-based modeling^14,15^, (iii) poor generalization with limited labelled data^13,20^, or (iv) excessive computational complexity that prevents real-time deployment^7,26^. Furthermore, efficiency is attained by lightweight models like Kitsune^17^, but robustness against intricate multi-stage attacks is compromised. These shortcomings underscore the need for an intrusion detection system that concurrently guarantees scalability, real-time applicability, adaptive ensemble fusion, and efficient temporal and attention modeling. In order to overcome these obstacles, this paper suggests HAMC-ID, which combines a dynamic attention-driven meta-classifier with a variety of base classifiers to provide both robustness and adaptability across several benchmark datasets.

## Proposed model

In order to effectively detect cyberattacks, this work presents HAMC-ID, a unique, lightweight ensemble model that blends a neural attention-based meta-classifier with conventional base learners. The proposed approach is designed to support both binary and multiclass classification scenarios and exhibits good performance over a range of intrusion types. The data preprocessing step, which initiates the HAMC-ID pipeline, consists of several important steps such initial cleaning, missing value resolution, categorical attribute label encoding, and feature scaling using Min-Max normalization. The most important features are recognized and retained with the Chi-Square (χ^2^) statistical test, and the dimensionality is decreased using PCA. To mitigate class imbalance issues, SMOTE is used to enhance the HAMC-ID generalizability across minority classes and is used to train the data also.

Base classifiers—LR, ET, and XGBoost—are given the refined features following preprocessing. Each classifier learns from the input data on its own. The meta-feature set is created by combining the results of these underlying models, such as logits, entropy, and prediction confidence. Before generating the final choice, this set is then sent to an attention-based BiLSTM neural meta-classifier, which dynamically allocates importance to each base model’s predictions.

**Fig. 1:**
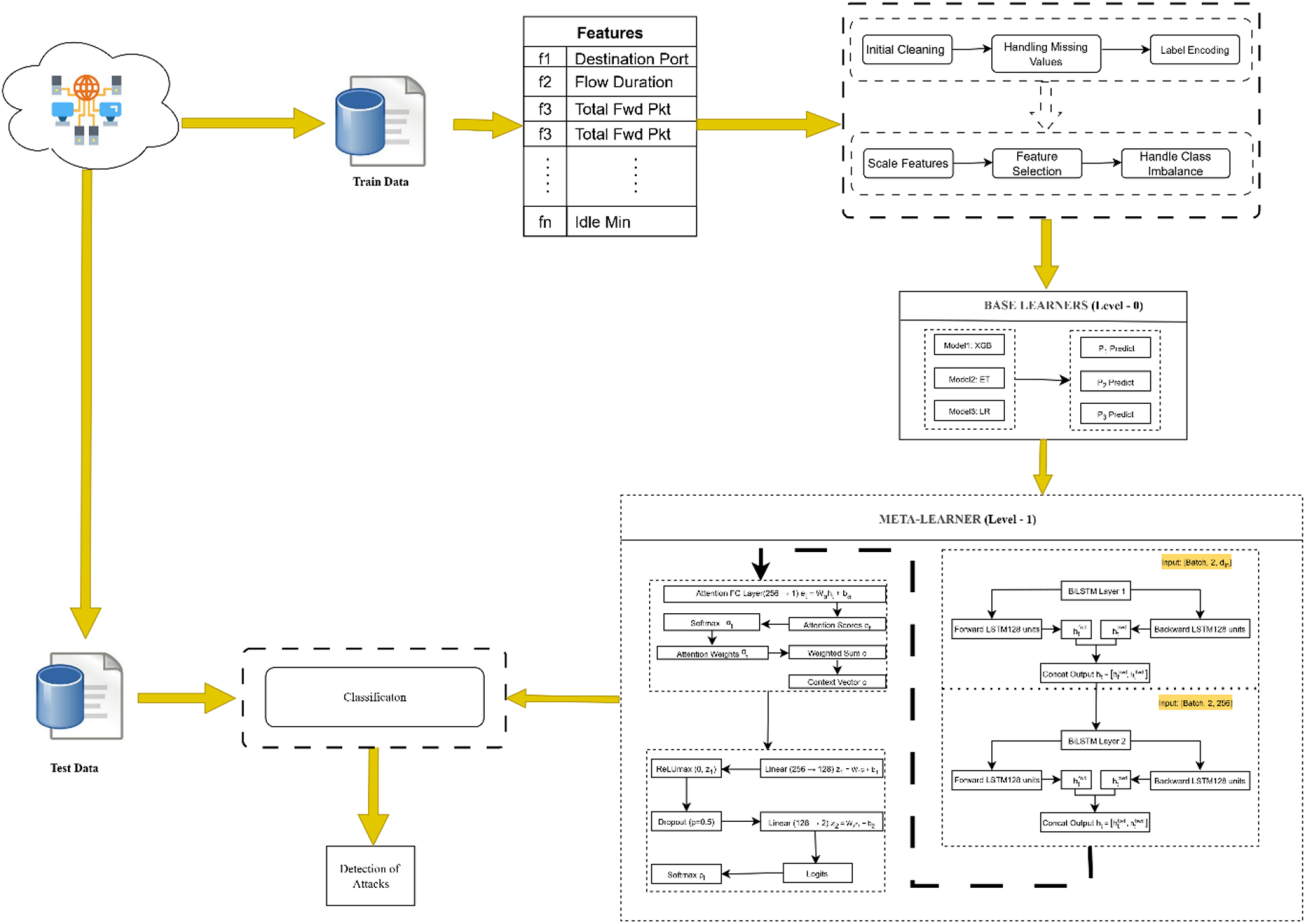
Proposed model.

The HAMC-ID overall structure is based on the 2-level stacking ensemble style, as shown in Fig. 1.

**Fig. 2:**
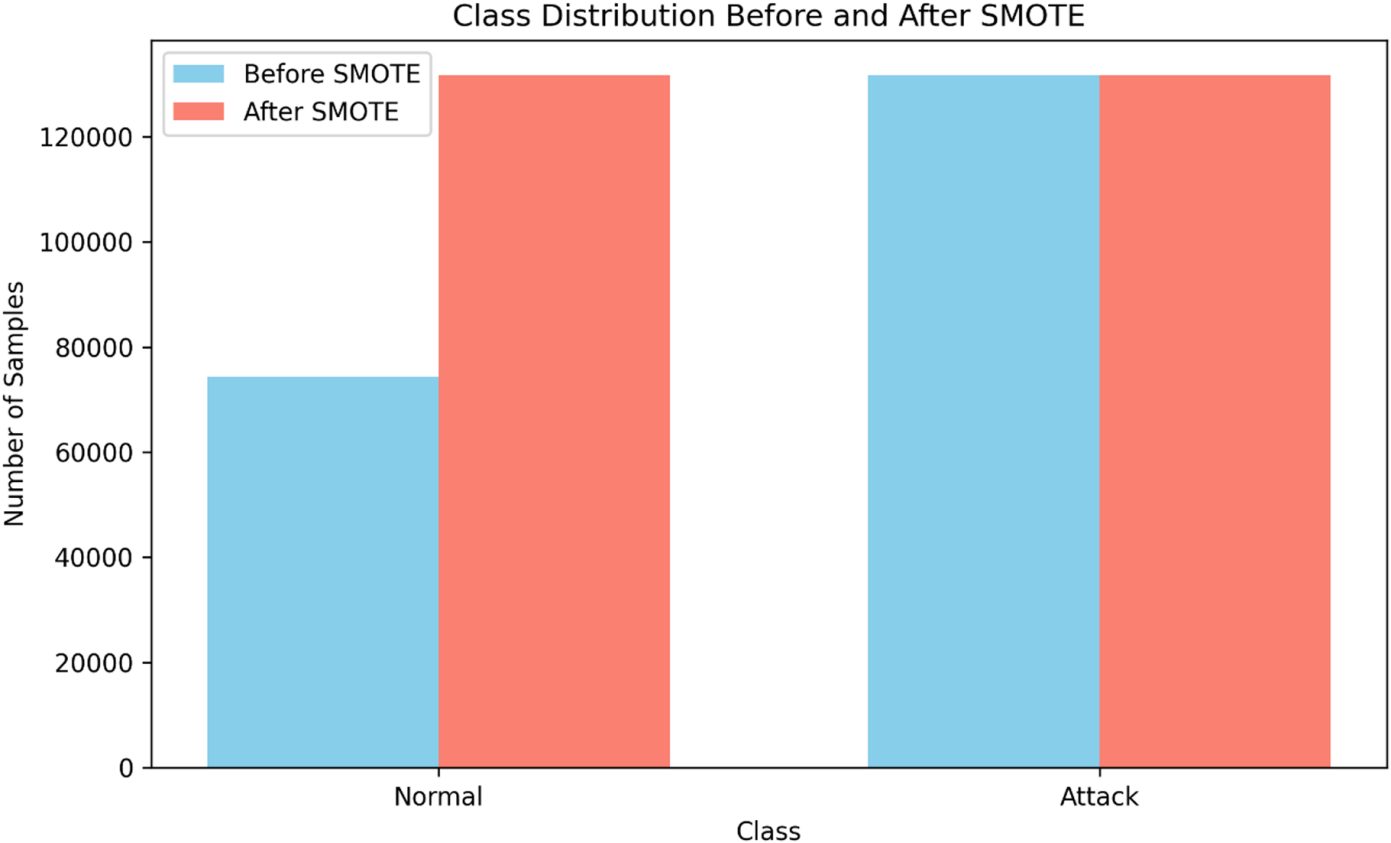
Class distribution before and after SMOTE.

Level-0 (Base Learners): Three distinct machine learning classifiers make up this layer: Logistic Regression (LR), Extra Trees Classifier (ET), and XGBoost. Because of their compatible decision-making processes and low computing overhead, these classifiers were specifically selected. The training dataset undergoes significant preparation, including label encoding, feature scaling using Min-Max normalization, dimensionality reduction using a combination of Principal Component Analysis in addition Chi-Square feature selection, and missing value imputation. The underlying class inequality is addressed through the Synthetic Minority Oversampling Technique, as illustrated in Fig. 2, which displays the balanced class distribution that emerges from its use. Each classifier is trained independently using this meticulously balanced and pre-processed dataset. During training, each base model generates significant meta-features. During training, each base model generates crucial meta-features—including logits, class confidence ratings, and entropy—which are then fed into the meta-classifier.

Level 1 (Meta-Learner): An attention mechanism and a fully connected classification module follow an attention-enhanced neural network constructed upon BiLSTM layers. The BiLSTM treats the stacked meta-features from the base classifiers as temporal sequences, thereby capturing inter-model dependencies and sequential relationships. By dynamically allocating weights to base model outputs, the attention mechanism emphasizes more reliable predictions, enabling the meta-classifier to adaptively prioritize informative signals. This adaptive weighting constitutes a significant advancement over static ensemble methods, enhancing the system’s generalization capability across diverse attack patterns.

For training, the UNSW-NB15 dataset is partitioned into 80:20 splits for training and testing to prevent data leakage, while the meta-classifier is trained on meta-features extracted from base-model predictions during training. A similar protocol is applied to CICIDS2017 to ensure consistency and fair evaluation across benchmarks. The testing sets remain strictly isolated until final evaluation, ensuring robust and unbiased performance assessment.

By using attention to enable dynamic and context-sensitive fusing of base classifier outputs, the suggested HAMC-ID architecture improves on conventional stacking ensembles. Increased accuracy and robustness result from this, especially in situations involving complicated or unbalanced intrusion detection.

The suggested HAMC-ID architecture was made scalable and reproducible by utilizing popular Python libraries for all implementation and testing, such as Scikit-learn, XGBoost, and Pandas.

### Preprocessing

A crucial part of the suggested HAMC-ID model is the preprocessing phase. To determine whether the UNSW-NB15 and CICIDS2017 datasets were appropriate for machine learning tasks, it first underwent exploratory data analysis (EDA). Among the several features that comprise the dataset are IP addresses, protocol kinds, flow durations, and packet statistics. A range of data types are also included, such as categorical variables (object), floating-point values (float64), and integers (int64).

Categorical elements like protocol, service, and state were converted into machine-understandable numerical representations via label encoding. The encoder defines a mapping function for a collection of distinct labels where L = {l_1_, l_2_,…., l_n_}.

$${\text{f}}({\text{I}}_{{\text{i}}} ) = {\text{i}} - 1\,\;\;\forall {\text{i}} \in [1,{\text{n}}].$$

By giving the models, the ability to understand non-numeric data, this transformation makes it easier to apply supervised learning. For binary classification, the target variable label—which differentiates between harmful and benign network flows—is kept.

The dataset was then checked for null entries, duplicate records, missing values, and NaNs. In order to minimize possible noise and bias during model training, these discrepancies were resolved using suitable imputation approaches. For numerical columns with missing values in particular, mean imputation was applied.

For the input features’ unequal scaling, all numerical values were converted into the [0,1] [0,1] range with Min-Max normalization. The formal definition of this normalization and was determined using Eq. (1)^13^:

1 $$\:\:{x}^{{\prime\:}}=\frac{x-\text{min}\left(x\right)}{\text{max}\left(x\right)-\text{min}\left(x\right)}$$

where the normalized value is denoted by $\:x$′ and the original feature value by $\:x$′. By taking this step, the learning process is kept from being dominated by features with large magnitudes and convergence stability is improved.

The identification of pertinent features is a critical phase in the HAMC-ID pipeline that comes after data preprocessing. In addition to lowering the computational cost of training the model, reducing the amount of input characteristics also enhances interpretability and reduces the risk of overfitting. To guarantee both statistical relevance and feature space compactness, the suggested model employs a 2-stage feature selection method.

First, the most informative features with regard to the class label (normal vs. intrusive) are found using the Chi-Square ($\:{\chi\:}^{2}$) test, is a statistical method to evaluate the association between categorical variables by comparing the observed and expected occurrences. An eventuality table is created for every feature-label pair, and the Chi-Square score is determined using Eq. (2):

2 $$\:{\chi\:}^{2}=\sum\:\frac{({{O}_{i}-{E}_{i})}^{2}}{{E}_{i}}$$

where the expected frequency for a certain cell in the eventuality table is denoted by means of $\:{E}_{i}$ and the observed frequency by $\:{O}_{i}13$. This is how the expected value is calculated using Eq. (3):

3 $$\:{E}_{i}=\:\frac{\left(Row\:Total\right)\times\:\left(Column\:Total\right)}{Grand\:Total}$$

**Fig. 3:**
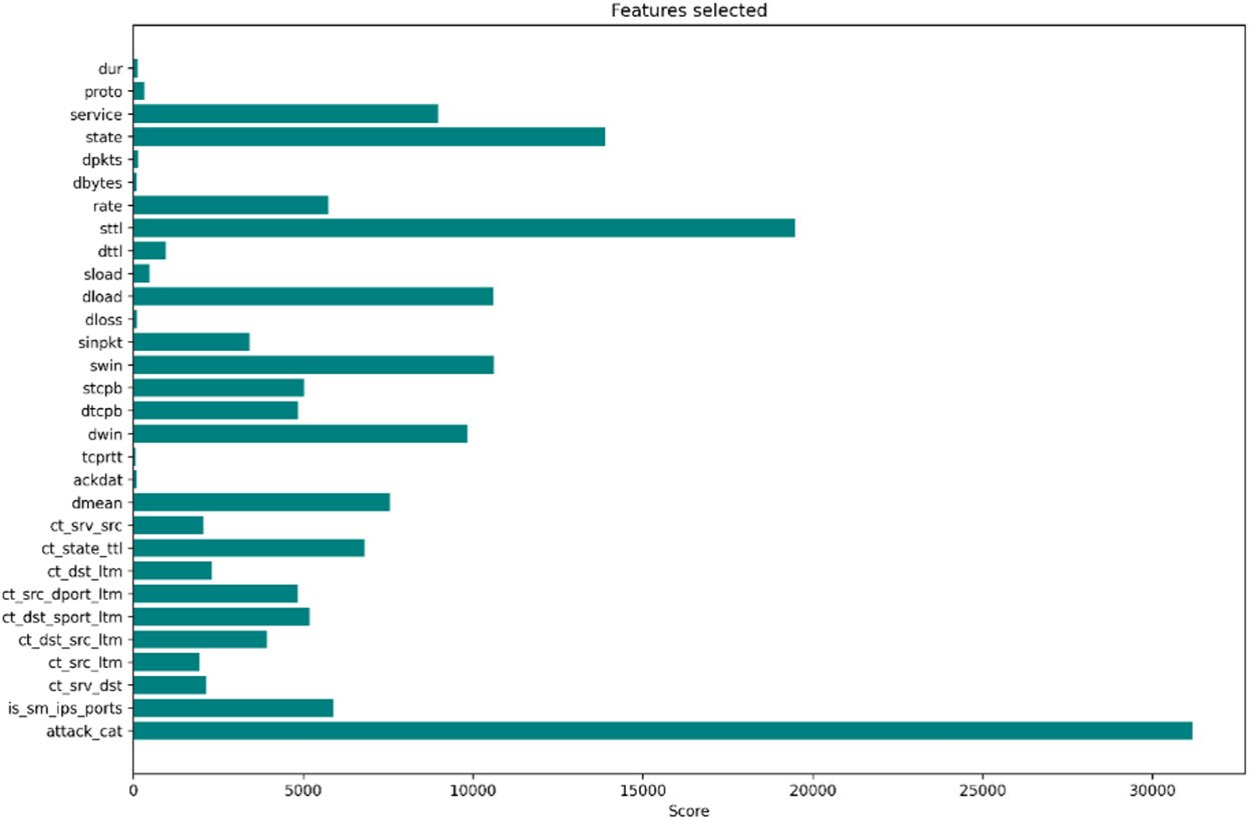
Features selected.

The observed and anticipated frequencies are almost similar when a feature is label-independent, which lowers the χ^2^ score. Features with greater χ^2^ values, on the other hand, show a stronger reliance on the target variable. The top 30 features, as indicated in Fig. 3, were selected for training Logistic Regression, XGBoost, and Extra Trees in the proposed implementation based on Chi-Square scores.

To further minimize dimensionality and avoid multicollinearity among the selected features, PCA was employed. PCA keeps the components that maintain the greatest variance after converting the input characteristics into an orthogonal basis. This model improved training speed and generalization by reducing the 30 Chi-Square chosen features to 20 principal components.

Apart from the filter-based approach, an embedded selection methodology based on the XGBoost classifier was also used to assess the feature relevance. When a feature is utilized to split the data during training, XGBoost measures the development in loss decrease using the Gain metric. The Gain at a decision node is calculated using Eq. (4) as follows:

4 $$\:\text{G}\text{a}\text{i}\text{n}\:=\frac{1}{2}\left[\frac{{G}_{L}^{2}}{{H}_{L}+\lambda\:}+\frac{{G}_{R}^{2}}{{H}_{R}+\lambda\:}-\frac{{({G}_{L}+{G}_{R})}^{2}}{{H}_{L}+{H}_{R}+\lambda\:}\right]-\gamma\:$$

Here, G_L_ and_L_ G_R_ denote the sum of first-order gradients (i.e., partial derivatives of the loss) for the left and right child nodes, respectively. H and H_R_​ denote the corresponding second-order gradients (Hessians). The parameter λ acts as an L_2_ regularization term, controlling the complexity of leaf nodes, while γ penalizes the addition of new leaves, preventing overfitting^14^. Higher Gain features are thought to be more important and have a greater impact on lowering model error. Despite the exploration of this embedding technique, the Chi-Square + PCA combination was kept in the final model due to its effectiveness and interpretability.

SMOTE was employed on the training data to balance the distribution of attack and benign occurrences, therefore enhancing the HAMC-ID ability towards acquire from minority class samples. This approach avoids biased learning and improves the model’s detection of rare intrusion types by producing false samples for minority classes, which results in a more balanced distribution. Feature transformation is followed by feeding the updated data into the proposed two-level stacking architecture. At Level 0, XGBoost, Extra Trees and Logistic Regression are trained independently as base classifiers. Meta-features (logits, entropy, and confidence scores) are processed by a BiLSTM-based attention-driven meta-classifier at Level 1 in order to learn adaptive fusion of base model outputs are saved. Through this dynamic learning technique, the model can improve overall intrusion detection accuracy and generalize across a wide variety of assault types.

### Base learning

At Level-0, the suggested HAMC-ID architecture makes use of three different base classifiers: XGBoost, ET, and LR. A variety of learning paradigms, including bagging-based, boosting-based, and linear, are represented by these classifiers, which were selected due to their low variance, computational efficiency, and complementing decision-making abilities. Independent training of these models improves generalization to a variety of assault types and increases ensemble diversity.

Logistic regression, Using the sigmoid activation function, the discriminative linear model known as logistic regression calculates the using Eq. (5) as follows conditional probability of a binary outcome:

5 $$\:P\left(y=1|x\right)=\frac{1}{1+{e}^{-({w}^{T}x+b)}}$$

where the learnt weight vector is denoted as $\:w\:$∈$\:\:\mathbb{R}$^d^, the bias is term as b ∈$\:\:\mathbb{R}$, and the input feature vector as $\:x\:$∈$\:\:\mathbb{R}$^d^. HAMC-ID is enhanced with Maximum Likelihood Estimation, which reduces the binary cross-entropy loss, as well-defined in (6).:

6 $$\:{\mathcal{L}}_{LR}=-\frac{1}{n}\sum\:_{i=1}^{n}\left[{y}_{i}\:log\right({\widehat{y}}_{i})+\left(1-{y}_{i}\right)\:log(1-{\widehat{y}}_{i}\left)\right]$$

LR is praised for its interpretability and low variance, and it works especially well for datasets that are linearly separable^30^.

Classifier for Extra Trees (ET), Two layers of randomization are added by the Extra Trees classifier, which is a randomized ensemble of decision trees: Without bootstrapping, it makes use of the complete training set. At each split, it chooses features’ cut-points at random. Each tree in the ensemble f_k(x) casts a vote for a particular input x, then the majority vote determines the final prediction using Eq. (7):

7 $$\:F\left(x\right)=mode({f}_{1\:}\left(x\right),{f}_{2}\left(x\right),\dots\:,{f}_{K}\left(x\right))$$

Because of their resistance to noise and variance reduction, extra trees frequently perform better than conventional decision trees. In a stacking framework, their non-deterministic character enhances ensemble diversity, which is advantageous.

XGBoost is a powerful ensemble learning algorithm that incrementally builds decision trees to minimize a given loss function. The HAMC-ID is trained iteratively, and at each stage t, the general objective function is defined as

8 $$\:{\mathcal{L}}^{\left(t\right)}=\sum\:_{i=1}^{n}l\left({y}_{i},{{\widehat{y}}_{i}}^{\left(t\right)}\right)+\sum\:_{k=1}^{t}{\Omega\:}\left({f}_{k}\right)$$

Where $\:l\left({y}_{i},{{\widehat{y}}_{i}}^{\left(t\right)}\right)\:$denotes the loss for prediction$\:\:{{\widehat{y}}_{i}}^{\left(t\right)}$​, then $\:{\Omega\:}\left({f}_{k}\right)$ is a regularization term that discourages overly complex trees by penalizing their structure.

Each function $\:{f}_{k} \in \mathcal{\:}\mathcal{F}$ represents a regression tree. The regularization term $\:{\Omega\:}\left(f\right)$ controls model complexity with Eq. (9):

9 $$\:\:{\Omega\:}\left(f\right)=\:{\gamma\:}^{T}+\frac{1}{2}\:\lambda\:\sum\:_{j=1}^{T}{w}_{j}^{2}$$

where T denotes the number of leaves, $\:{w}_{j}$ is the weight assigned to the j^th^ leaf, γ is a regularization coefficient controlling the cost of adding new leaves, and λ applies L2 regularization on leaf weights. XGBoost strength lies in its capability to capture nonlinear associations in addition feature relations while keeping generalization through strong regularization approaches^31^, makes it ideal for imbalanced and high-dimensional intrusion detection datasets.

### Meta-learning: attention-based BiLSTM

The Level-1 meta-classifier of the HAMC-ID architecture is a BiLSTM network with attention augmentation designed to dynamically integrate the predictions of the Level-0 classifiers. This model learns to allocate adaptive weights to each base learner based on confidence and uncertainty measurements, as opposed to statically averaging or voting on outputs.

#### Meta-feature construction

For each sample $\:{x}_{i}$ the following meta-features are extracted from each base model m∈ {LR, ET, XGB}:

*Logits*: $$\:{l}_{i}^{m}=\text{l}\text{o}\text{g}\left(p\right(y|{x}_{i\:};m))$$

*Confidence*: $$\:{c}_{i}^{m}=\text{m}\text{a}\text{x}\left(p\right(y|{x}_{i\:};m))$$

*Entropy*: $$\:{e}_{i}^{m}=-{\sum\:}_{k}pk\text{log}pk$$

These are concatenated to form the meta-feature vector $\:{\varnothing\:}_{i}^{m}=[{l}_{i}^{m},{c}_{i}^{m},{e}_{i}^{m}]$.The final meta-feature tensor is:

Φ∈$\:\mathbb{R}$^n×T×f^, where T = 3 (base models), f = 3 (features/model).

#### BiLSTM + attention aggregation

The BiLSTM processes each temporal sequence Φ_i_ to generate hidden states $\:{h}_{t}$ ∈ $\:\mathbb{R}$^h^ for each timestep t:

$$\:{h}_{t}$$ =BiLSTM(Φ_t_;θ).

An *attention mechanism* computes a weight $\:{\alpha\:}_{t}\:$for each hidden state using Eq. (10):

10 $$\:{\alpha\:}_{t}=\:\frac{\text{e}\text{x}\text{p}\left({w}_{a}^{T}\:tanh\right({W}_{h}{h}_{t}\left)\right)}{\sum\:_{j=1}^{T}\text{e}\text{x}\text{p}\left(tanh\right({W}_{h}{h}_{j}\left)\right)}\:$$

$$\:c=\:{\sum\:}_{t=1}^{T}{a}_{t}{h}_{t}$$.

The final context vector $\:c$ captures the weighted contributions of each base model’s outputs. It is passed to a fully connected layer for prediction:

$$\:\widehat{y}=softmax\left({W}_{c\:}\right[c;{h}_{t}\left]\right)$$

This approach increases the model’s resilience to individual base model faults by allowing it to concentrate more on accurate predictions. By serving as an adaptive gating function, the attention mechanism strengthens HAMC-ID’s resistance to ambiguous or noisy signals.

## Experimental results and evaluation

The performance of the HAMC-ID was evaluated with the UNSW-NB15 dataset, which gives a wide-ranging and contemporary benchmark for IDS. Disparate previous datasets, UNSW-NB15 mirrors up-to-date network behaviours, includes updated protocol usage, and presents a balanced distribution of both benign and malicious traffic. The dataset is organized into ten distinct classes: one class corresponds to legitimate traffic, while the remaining nine—that is Analysis, Denial of Service, Generic, Backdoor, Shellcode, Exploits, Reconnaissance, Fuzzers, and Worms—represent various types of cyberattacks. In total, the dataset contains 42 features, encompassing both numerical and categorical attributes, enabling effective modelling of diverse attack patterns. The total samples used in this work are summarized in Table 2^32^.

**Table 2:** Class-wise distribution of UNSW-NB15.

Attack category	Training	Evaluation	Total
Normal	56,000	37,000	93,000
Generic	40,000	18,871	58,871
Exploits	33,393	11,132	44,525
Fuzzers	18,184	6062	24,246
DoS	12,264	4089	16,353
Reconnaissance	10,491	3496	13,987
Analysis	2000	677	2677
Backdoor	1746	583	2329
Shellcode	1133	378	1511
Worms	130	44	174
**Total**	175,341	82,332	257,673

This segment presents the valuation outcomes of individual classifiers alongside those of the HAMC-ID. The model is trained using both binary and multiclass classification strategies, and its performance is calculated across different dataset configurations to demonstrate its flexibility and usefulness. The performance of the HAMC-ID was further validated on the CICIDS2017 dataset, which has become one of the most widely used benchmarks for IDS research due to its realistic multi-protocol traffic and comprehensive attack coverage. Unlike earlier datasets, CICIDS2017 captures modern network behaviours, incorporating updated services, background traffic, and diverse attack vectors. The dataset is structured into eight attack categories—Brute Force, DoS, Botnet, PortScan, Web Attack, Infiltration, Heartbleed, and DDoS—alongside normal traffic, offering a multi-class evaluation setting. In total, the dataset comprises 80 flow-based features that cover statistical characteristics such as packet length, inter-arrival times, and flow duration, enabling detailed analysis of network behaviour. 4.1 **Experimental Setup**.

Experiments were performed on a workstation with Intel i7 (3.4 GHz), 16 GB RAM, and NVIDIA GTX 1660 GPU (Ubuntu 22.04, Python 3.10, Scikit-learn 1.3, PyTorch 2.0, XGBoost 1.7). Logistic Regression (L2, C ∈ {0.01, 0.1, 1, 10}), Extra Trees (200 estimators, max_depth ∈ {10, 20, None}), XGBoost (learning_rate ∈ {0.01, 0.1}, max_depth ∈ {3, 6, 10}, n_estimators ∈ {100, 200}), and GaussianNB (default) were tuned, while the PyTorch attention fusion used hidden size = 128, dropout = 0.3, Adam optimizer (lr = 0.001, batch = 128, 30 epochs). On this setup, base models trained in ~ 30 s (inference 0.6 s), whereas HAMC-ID required ~ 4354 s for training and ~ 9.1 s for inference. Reported inference times correspond to processing the full test set of size N.

### Binary classification result

Table 3 summarizes the binary classification results on the UNSW-NB15 dataset. HAMC-ID demonstrates consistently superior performance compared to its constituent base classifiers. The moderately lower performance of most individual models can be attributed to the dataset’s inherent class imbalance, which hampers their ability to effectively detect minority class instances. In contrast, HAMC-ID achieved a remarkable accuracy of 98.94%, underscoring the benefit of the proposed attention-based meta-classifier in handling such imbalance.

To further validate the robustness of the framework, we extended the evaluation to the CICIDS2017 dataset, with results presented in Table 4. Here as well, HAMC-ID consistently outperforms Logistic Regression, Extra Trees, and XGBoost across all metrics. Notably, HAMC-ID achieved an accuracy of 95.27%, reflecting its strong generalization ability across heterogeneous intrusion detection benchmarks. These findings collectively highlight that while individual classifiers are often susceptible to class disparity and dataset-specific limitations, the proposed HAMC-ID framework leverages attention-driven fusion to deliver more stable and reliable performance in diverse intrusion detection scenarios.

**Table 3:** Performance Metrics_UNSW-NB15 (Binary Classification).

Classifier	Class	Precision	Recall	F1 score	Accuracy (%)
Logistic Regression	Normal Intrusion	0.8671 0.9688	0.9476 0.9180	0.9056 0.9427	92.87
Extra Trees	Normal Intrusion	0.9559 0.9744	0.9547 0.9751	0.9553 0.9748	96.77
XGBoost	Normal Intrusion	0.9973 0.8331	0.6457 0.9990	0.7839 0.9086	87.15
HAMC-ID	Normal Intrusion	0.9807 0.9943	0.9900 0.9890	0.9853 0.9916	98.94

**Table 4:** Performance Metrics_ CICIDS2017 (Binary Classification).

Classifier	Class	Precision	Recall	F1 Score	Accuracy (%)
Logistic Regression	Normal Intrusion	0.983 0.5126	0.7765 0.9591	0.8693 0.6681	81.25
Extra Trees	Normal Intrusion	0.9904 0.6204	0.8552 0.9661	0.9178 0.7556	87.70
XGBoost	Normal Intrusion	0.8766 0.9977	0.9998 0.4258	0.9342 0.5969	88.68
HAMC-ID	Normal Intrusion	0.9950 0.8164	0.9460 0.9804	0.9698 0.8909	95.27

**Fig. 4:**
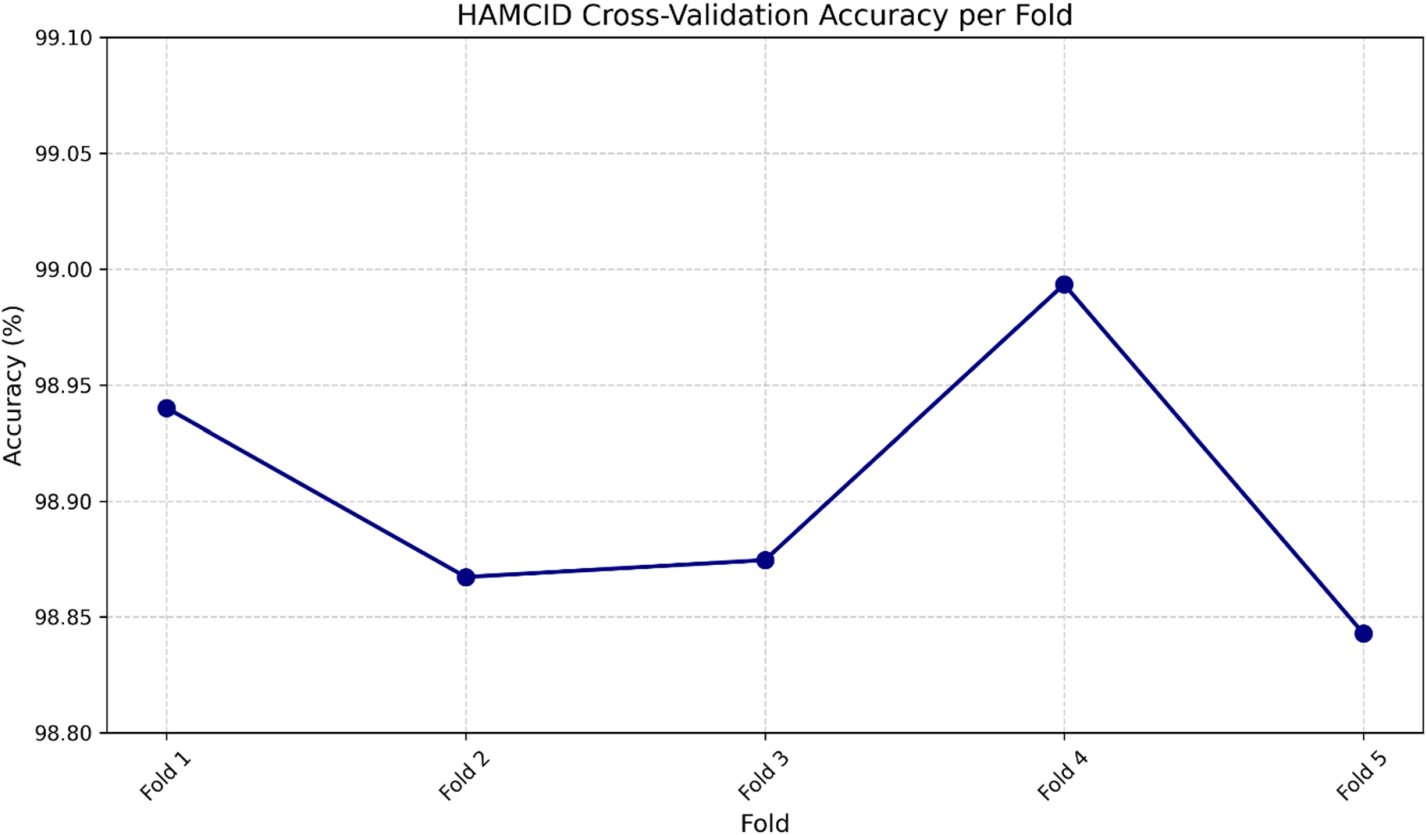
Cross validation accuracy.

To evaluate the strength and generalization ability of HAMC-ID, a five-fold cross-validation strategy was employed. In each fold binary classification accuracy is obtained, same is illustrated in Fig. 4, highlighting the model’s stability across different data partitions. The graph’s tight clustering of all accuracy scores within a small range graphically validates the model’s steady and reliable performance. With a mean accuracy of almost 98.90% over the five folds and a relatively low standard deviation of 0.05%, the HAMC-ID performance is not much impacted by means of the particular data partitions. Strong proof that the HAMC-ID model is not overfitting and can successfully generalize to unseen network data is provided by its stability, which is essential for a workable intrusion detection system. Furthermore, to ensure reproducibility, the repeated 5-fold cross-validation with five different random seeds (7, 21, 42, 84, 123). The results, summarized in Table 5, confirm that all models exhibit negligible variance across seeds, thereby demonstrating robustness and stability beyond a single random initialization.

**Table 5:** Cross-validation reproducibility across random seeds.

Seed	LR_mean	LR_std	ET_mean	ET_std	XGB_mean	XGB_std	HAMC-ID_mean	HAMC-ID_std
7	0.92397	0.00142	0.98812	0.00228	0.8728	0.0014	0.99886	0.00021
21	0.92401	0.00092	0.98957	0.00275	0.8751	0.0017	0.99934	0.00016
42	0.92399	0.00086	0.98533	0.00225	0.8705	0.0012	0.99863	0.00020
84	0.92381	0.00165	0.98897	0.00265	0.8736	0.0015	0.99917	0.00015
123	0.92393	0.00084	0.99068	0.00361	0.8712	0.0013	0.99890	0.00012

On the UNSW-NB15 dataset, HAMC-ID achieved strong classification performance, with the Normal class yielding a precision of 0.9807, recall of 0.9900, and F1-score of 0.9853, while the Intrusion class obtained precision of 0.9943, recall of 0.9890, and F1-score of 0.9916. Overall, HAMC-ID reached an accuracy of 98.94%, outperforming Logistic Regression (92.87%), Extra Trees (96.77%), and XGBoost (87.15%). These results indicate that the proposed model is particularly effective in handling class imbalance, where base classifiers showed reduced sensitivity to minority classes.

**Fig. 5:**
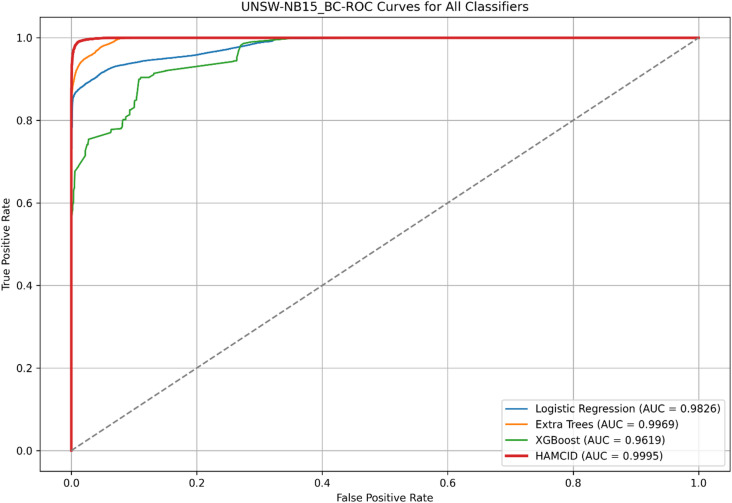
ROC Curvers for All Classifiers _UNSW-NB15 (Binary Classification).

**Fig. 6:**
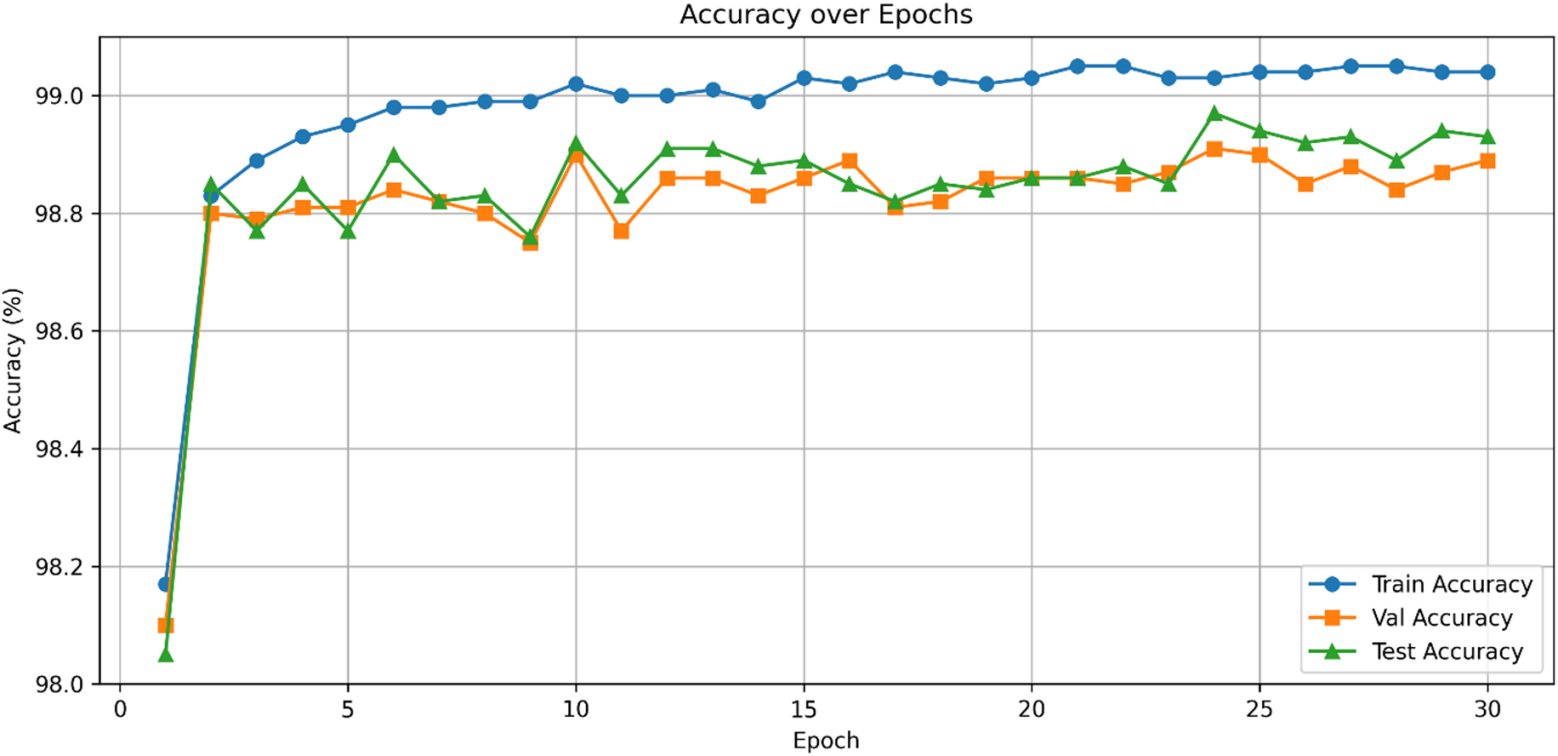
Train, Test and Validation accuracy curves across epochs_UNSW-NB15 (Binary Classification).

**Fig. 7:**
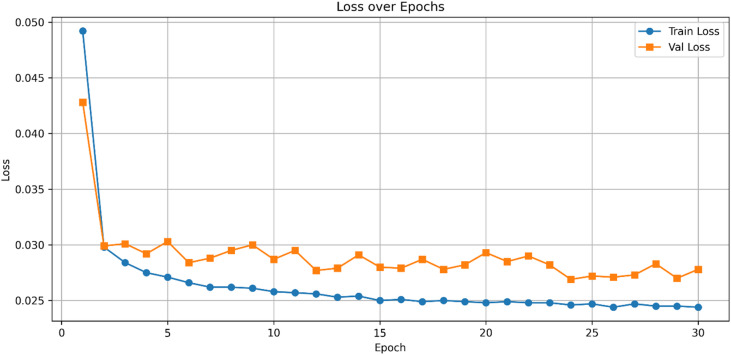
Train and validation loss curves across epochs_UNSW-NB15 (Binary Classification).

**Fig. 8:**
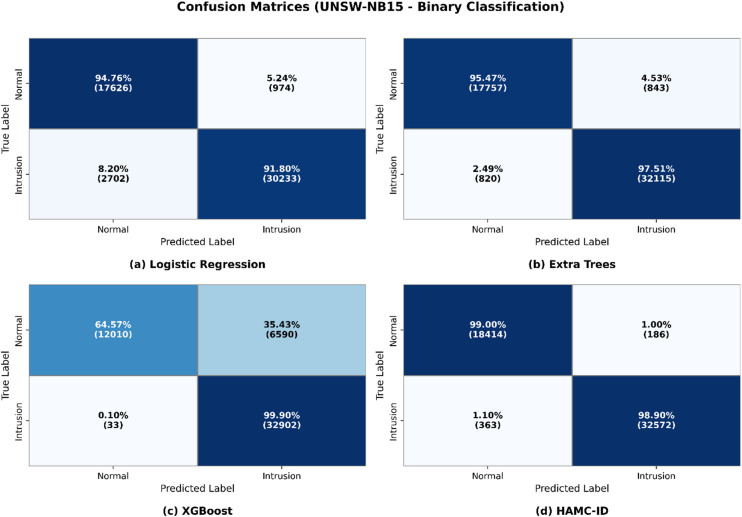
Confusion Matrix_UNSW-NB15 (Binary Classification).

Figure 5 presents the ROC curves for UNSW-NB15, where HAMC-ID achieved an AUC of 0.9995, compared to 0.9969 (Extra Trees), 0.9826 (Logistic Regression), and 0.9619 (XGBoost). The accuracy-over-epochs curves (Fig. 6) illustrate that HAMC-ID converges rapidly and stabilizes at ~ 98.9%, while the training and validation loss curves (Fig. 7) confirm consistent error minimization without evidence of overfitting. The confusion matrix (Fig. 8) further demonstrates reduced misclassification relative to base learners, underscoring the robustness of the proposed approach.

To assess generalization, we extended the evaluation to the CICIDS2017 dataset. HAMC-ID again outperformed baseline models, achieving an accuracy of 95.27%. For the Normal class, it reached precision of 0.9950, recall of 0.9460, and F1-score of 0.9698, while the Intrusion class yielded precision of 0.8164, recall of 0.9804, and F1-score of 0.8909. By comparison, Logistic Regression, Extra Trees, and XGBoost achieved accuracies of 81.25%, 87.70%, and 88.68%, respectively.

**Fig. 9:**
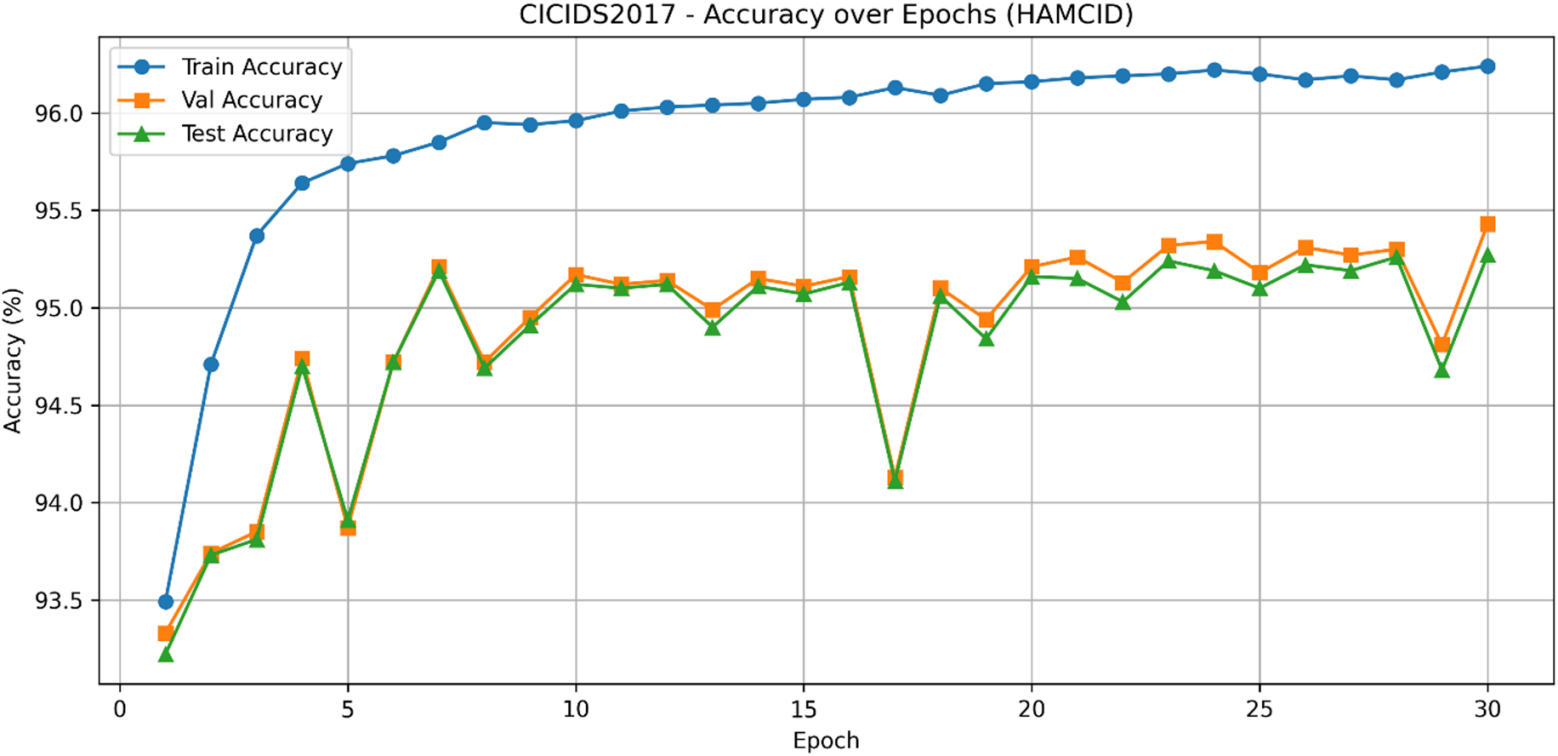
Train, Test and Validation accuracy curves across epochs_CICIDS2017(Binary Classification).

**Fig. 10:**
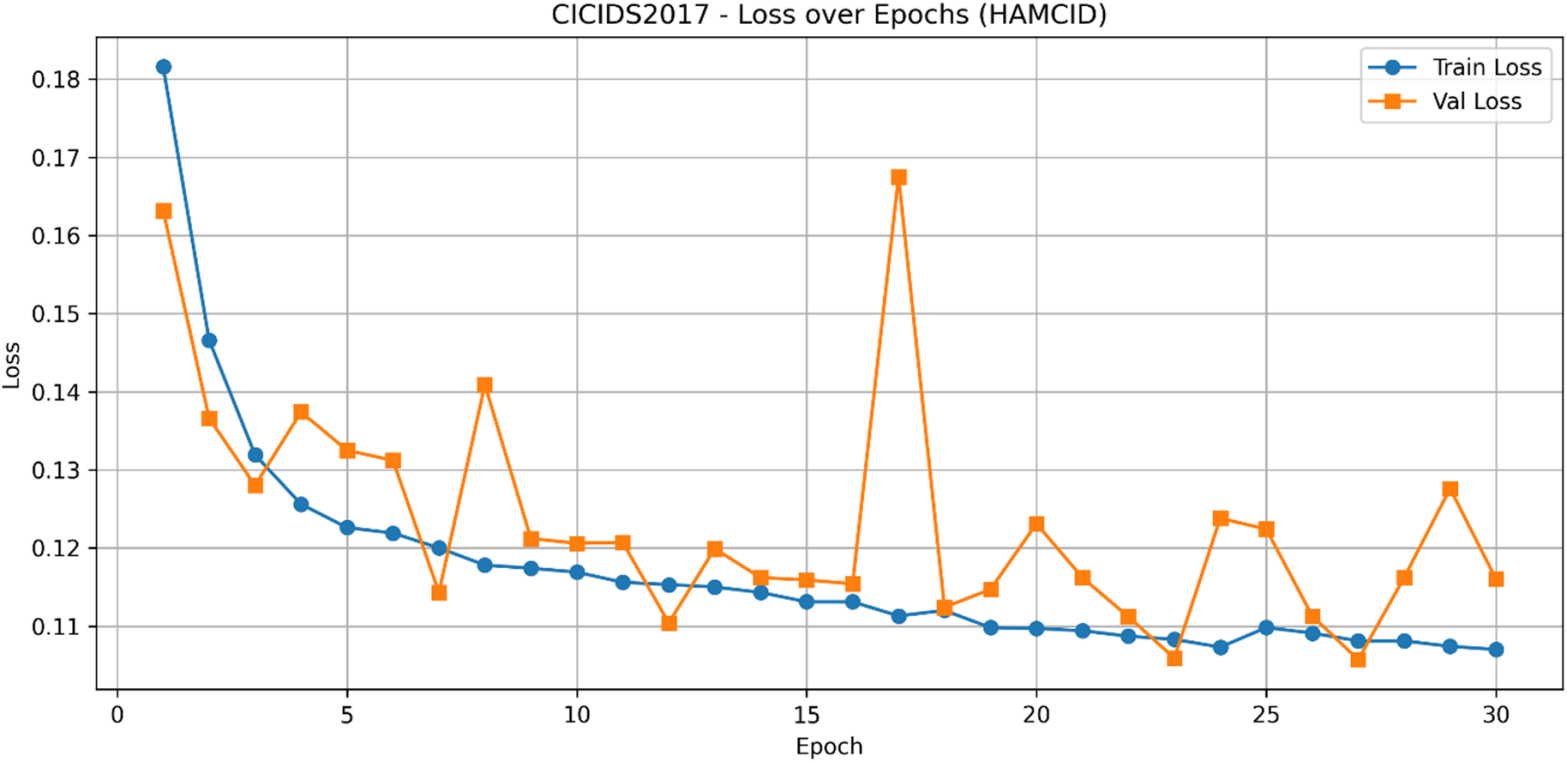
Train and validation loss curves across epochs_CICIDS2017 (Binary Classification).

**Fig. 11:**
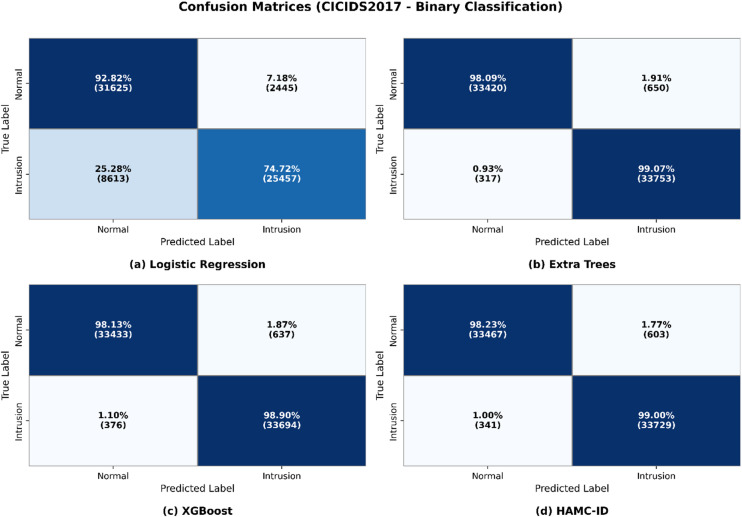
Confusion Matrix_CICIDS2017 (Binary Classification**)**.

**Fig. 12:**
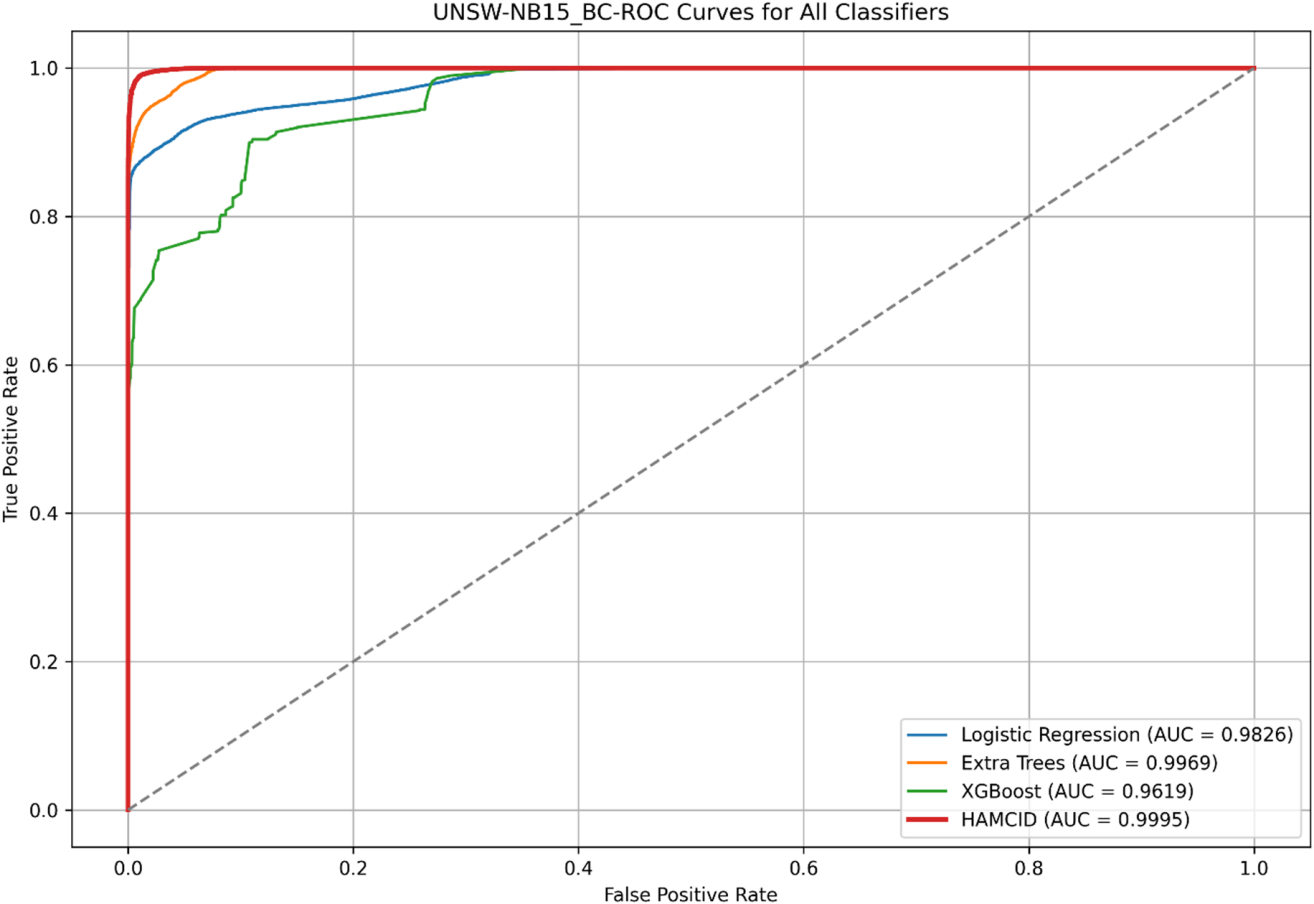
ROC Curvers for All Classifiers_CICIDS2017(Binary Classification).

The learning curves for CICIDS2017 (Figs. 9 and 10) show stable improvements in accuracy and loss convergence, consistent with the UNSW-NB15 findings. The confusion matrix (Fig. 11) indicates lower misclassification rates, particularly for intrusion detection, while the ROC curves (Fig. 12) confirm HAMC-ID’s superior discriminative capacity on this dataset.

Overall, the results across both datasets establish that HAMC-ID not only surpasses individual classifiers but also exhibits strong generalization, stability, and resilience to class imbalance, thereby validating its effectiveness for intrusion detection.

### Multiclass detection result

Both the UNSW-NB15 and CICIDS2017 datasets pose significant problems in the context of multiclass intrusion detection because of their intrinsic class imbalance and diverse assault characteristics. All classes, particularly those with limited representation, were difficult for conventional classifiers to generalize to. Logistic Regression showed a notably poor ability to predict minority classes like Worms and Shellcode for UNSW-NB15 (Table 6), producing F1-scores as low as 0.0285 and 0.1474. Even though ensemble-based models like XGBoost and Extra Trees achieved competitive results for the majority classes (Generic and Normal), they struggled against uncommon attacks like Worms and Backdoor. In CICIDS2017 (Table 7), Logistic Regression and Extra Trees also performed poorly in recognizing Web Attacks and Infiltration, with multiple classes receiving zero F1-scores. In contrast, XGBoost performed well on Benign and PortScan but poorly on low-frequency attacks.

**Fig. 13:**
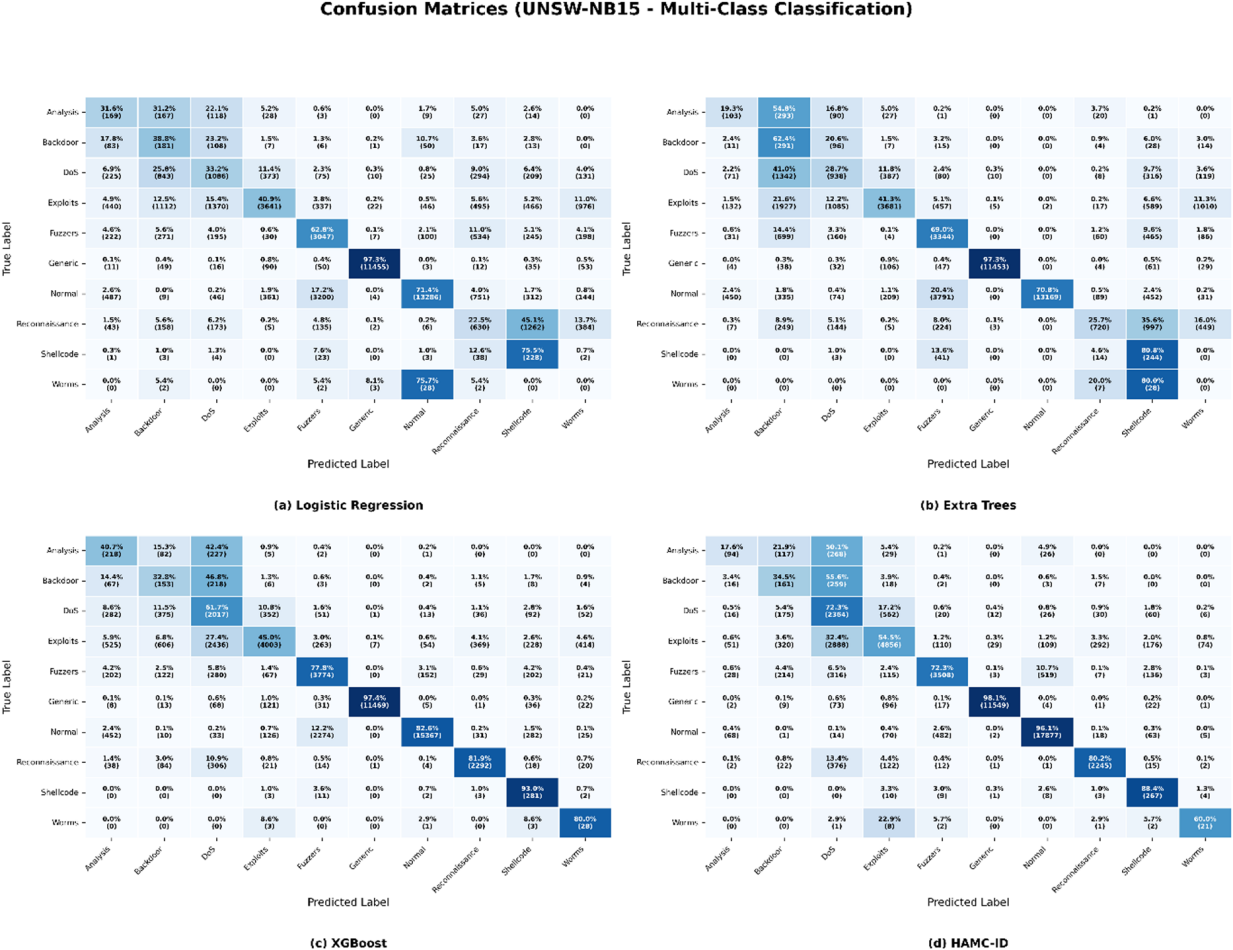
Confusion Matrix_UNSW-NB15 (Multiclass Classification).

**Fig. 14:**
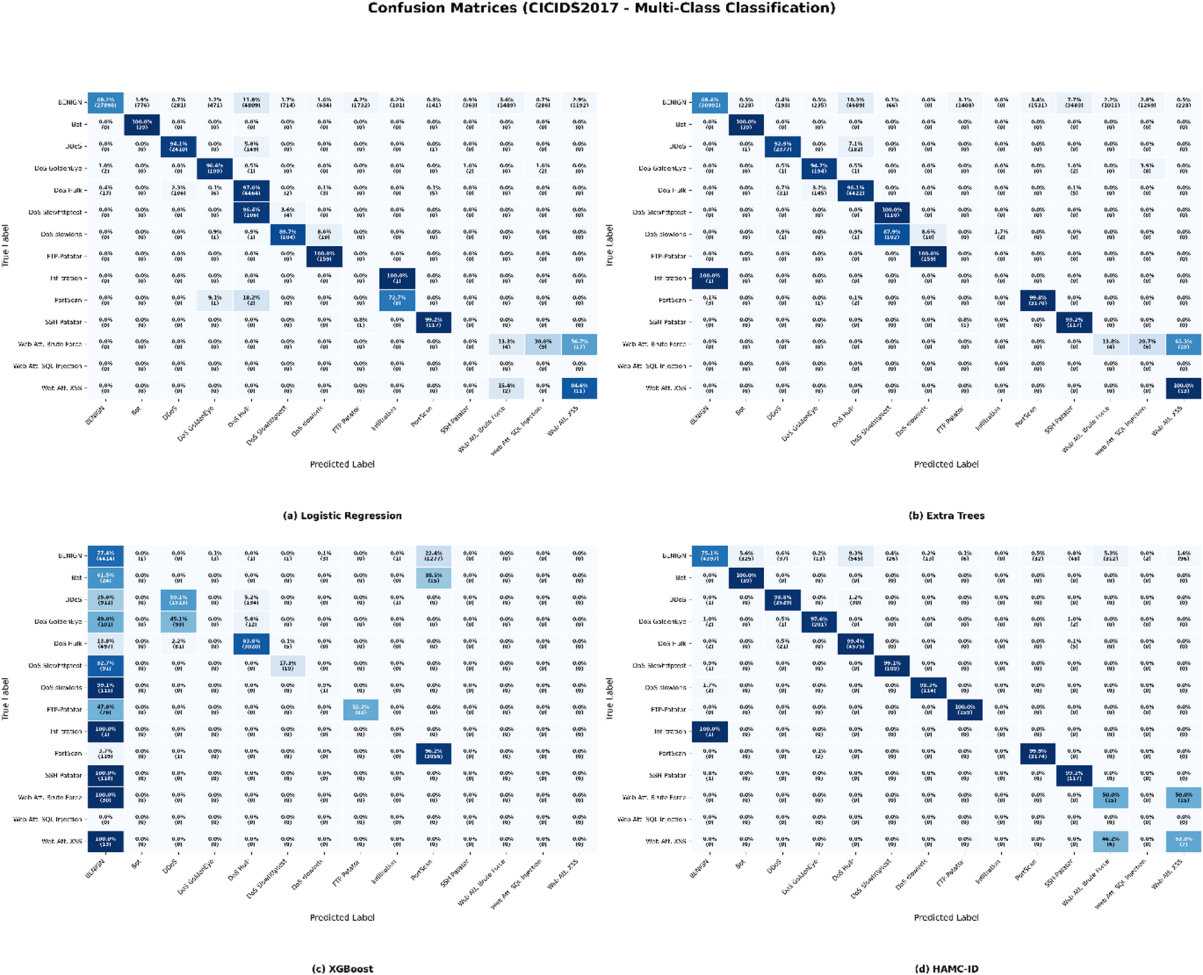
Confusion Matrix_CICIDS2017 (Multiclass Classification).

These limitations are further illustrated by the confusion matrices (Figs. 13 and 14), which demonstrate that traditional classifiers demonstrated substantial confusion between semantically related categories and frequently misclassified minority attacks. Logistic Regression and Additional Trees frequently misclassified Worms, Shellcode, and Analysis for UNSW-NB15. Logistic Regression and Extra Trees in CICIDS2017 confused DoS variations and Web Attacks, whereas XGBoost did a good job of separating dominant classes but had trouble with SSH-Patator and Infiltration.

However, in both datasets, the suggested HAMC-ID model showed noticeably better class-wise detection. HAMC-ID outperformed Logistic Regression (65.49%), Extra Trees (65.92%), and XGBoost (76.90%) on UNSW-NB15, achieving an overall accuracy of 83.33%. Notable gains were made for minority classes, including Shellcode (0.5086), Worms (0.2781), and Backdoor (0.2168). Likewise, HAMC-ID achieved 97.26% accuracy on CICIDS2017, outperforming XGBoost (91.64%), Extra Trees (73.74%), and Logistic Regression (68.39%). High F1-scores were obtained for DoS Hulk (0.9380), DoS Slowloris (0.9383), and PortScan (0.9947). Additionally, it improved detection for Web Attack – XSS (0.1069) and SSH-Patator (0.8269). HAMC-ID regularly beat baselines across all other minority classes, but infiltration was overlooked because of its extreme scarcity.

**Fig. 15:**
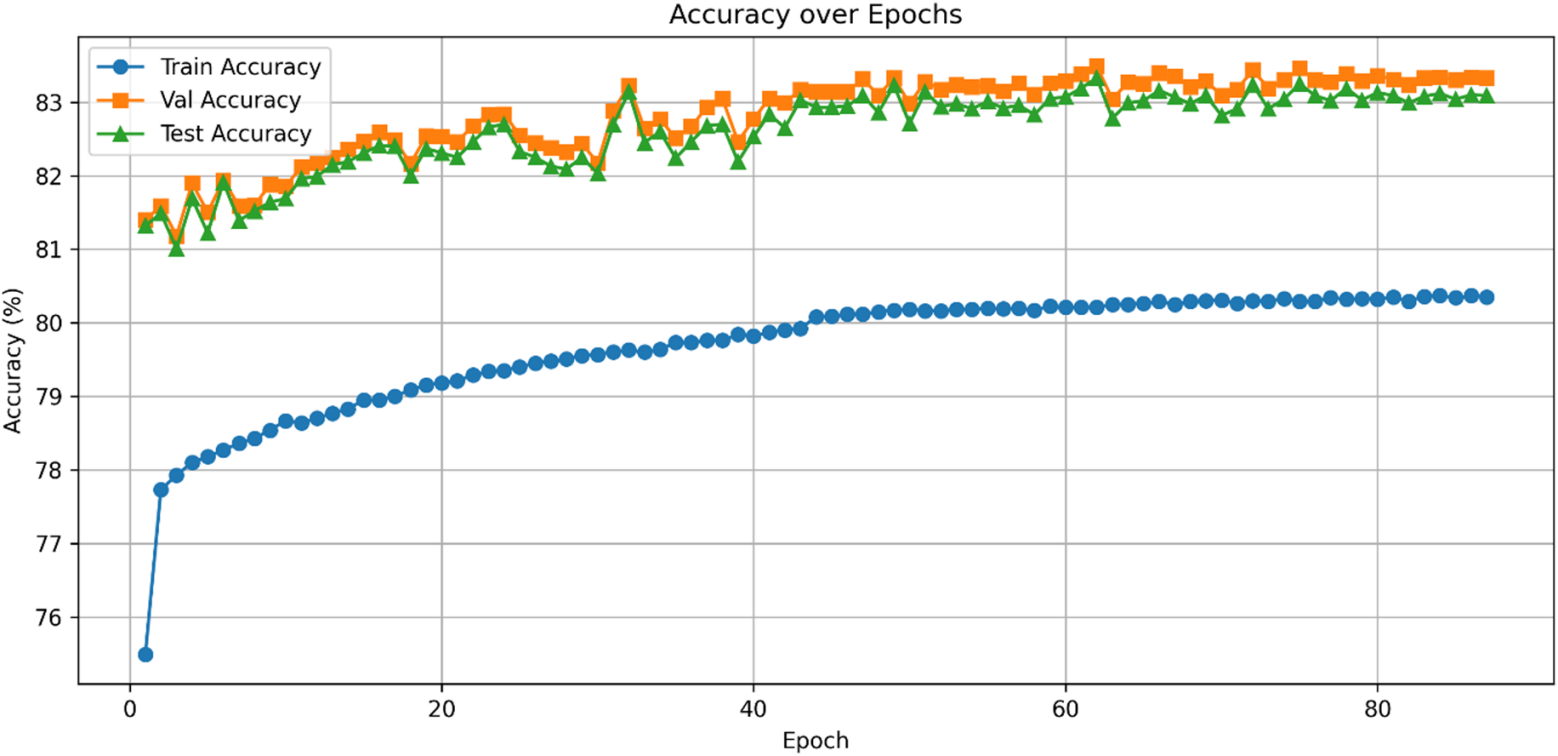
Accuracy over Epochs_UNSW-NB15 (Multiclass Classification).

**Fig. 16:**
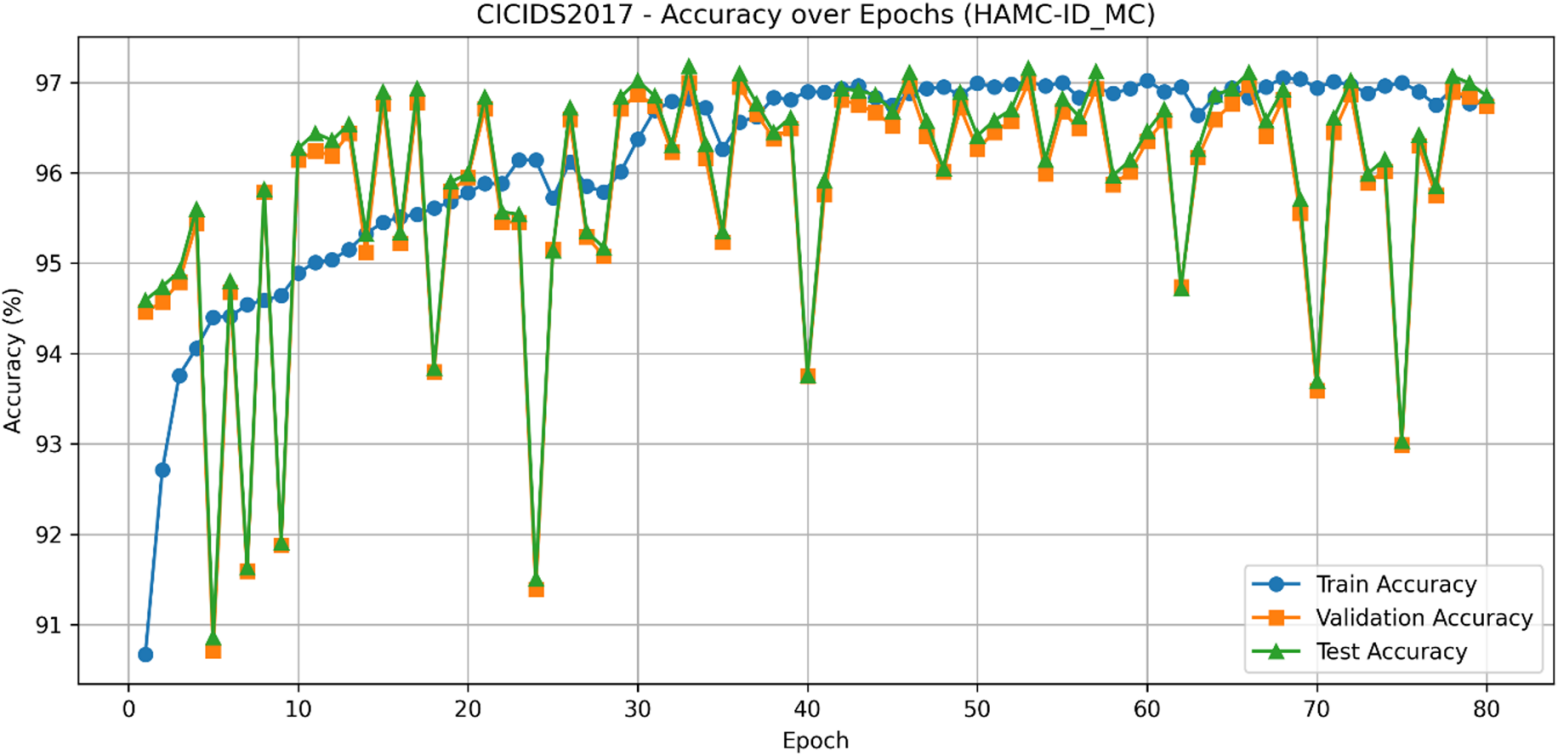
Accuracy over Epochs _CICIDS2017 (Multiclass Classification).

**Fig. 17:**
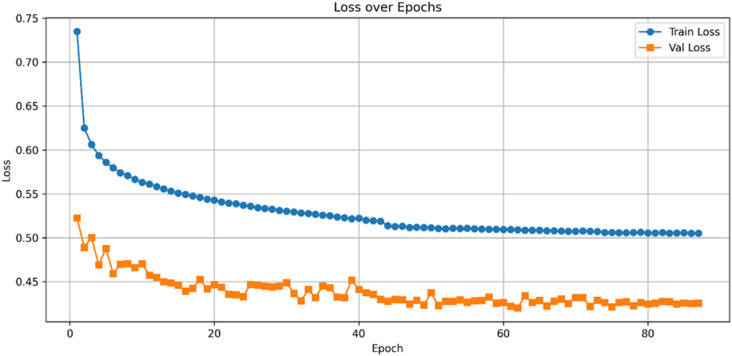
Loss over Epochs_UNSW-NB15 (Multiclass Classification).

**Fig. 18:**
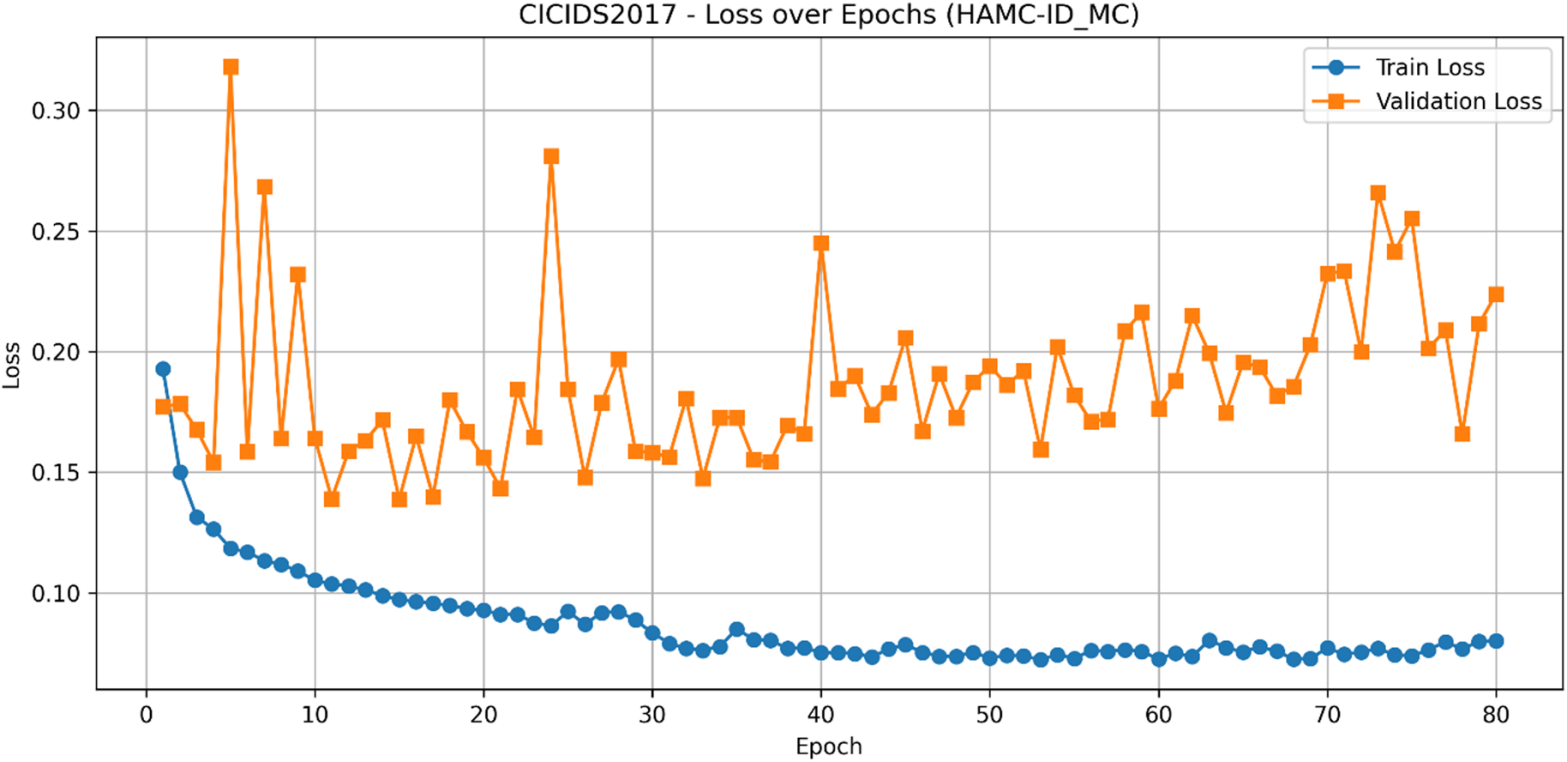
Loss over Epochs_CICIDS2017(Multiclass Classification).

**Fig. 19:**
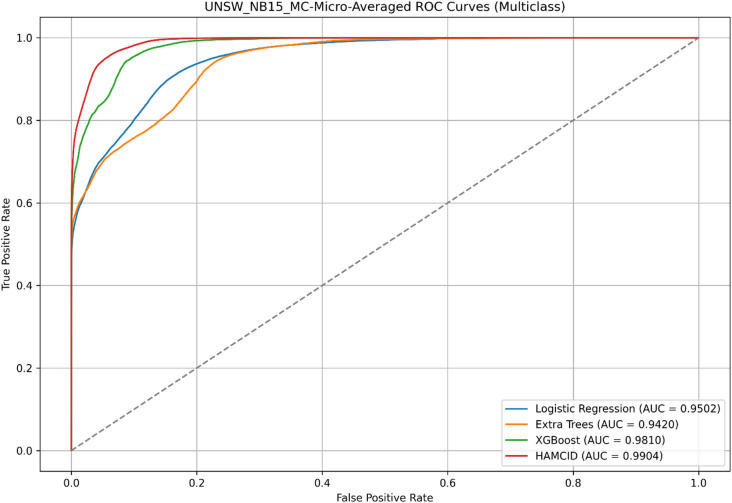
Micro-averaged ROC_UNSW-NB15 (Multiclass Classification).

**Fig. 20:**
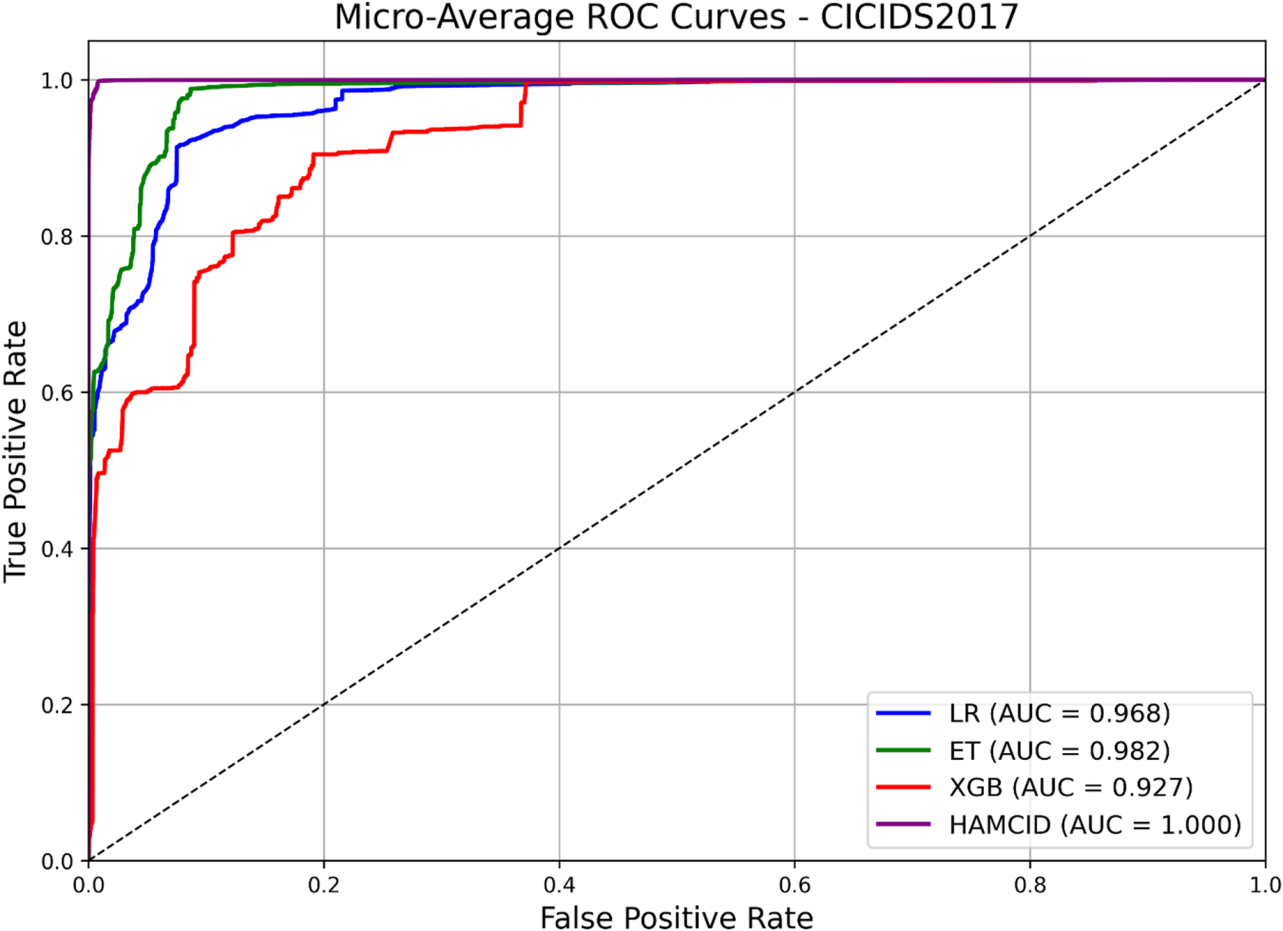
Micro-Averaged ROC_CICIDS2017 (Multiclass Classification).

**Fig. 21:**
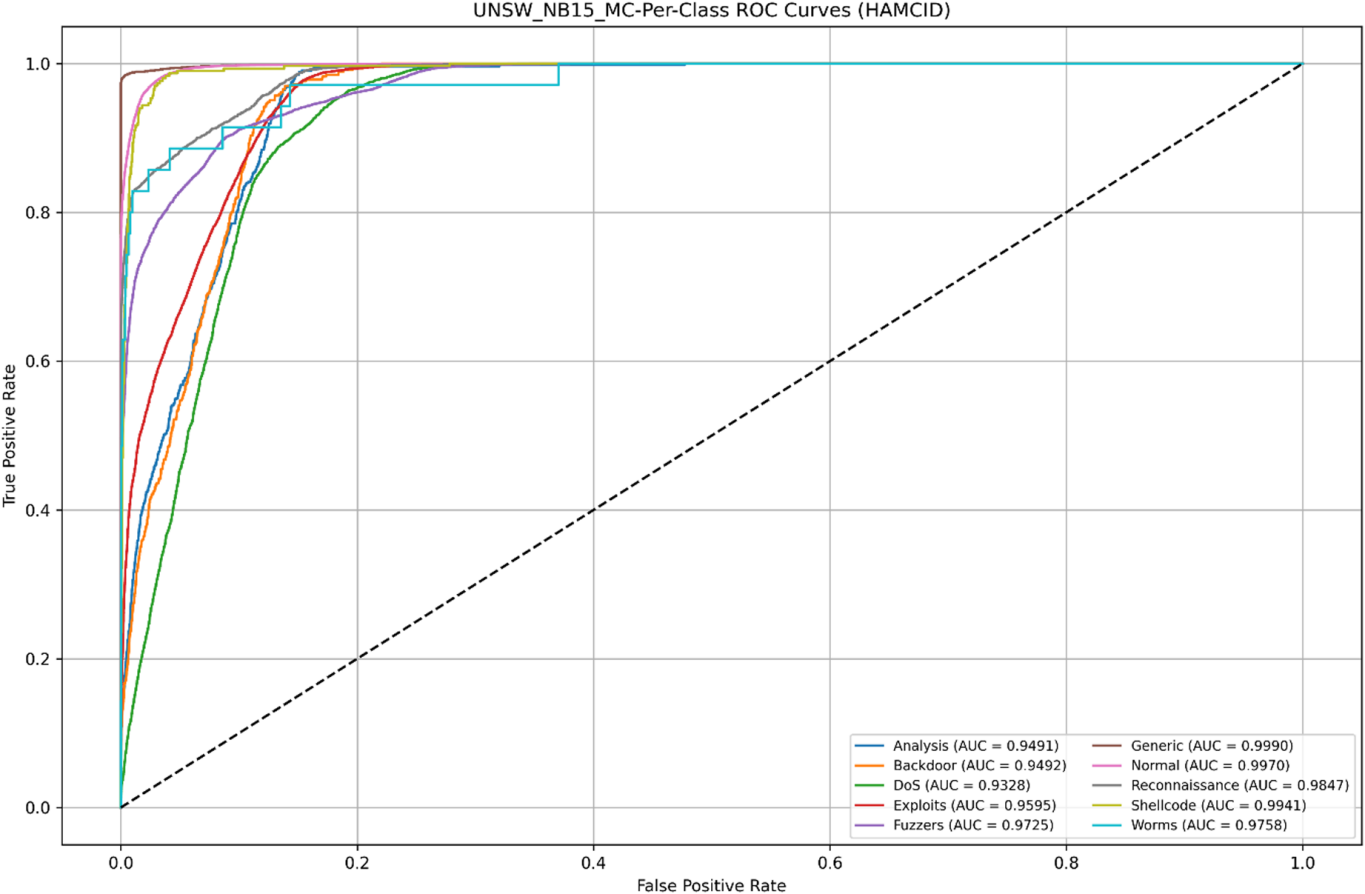
Per-Class ROC Curves _UNSW-NB15 (Multiclass Classification).

**Fig. 22:**
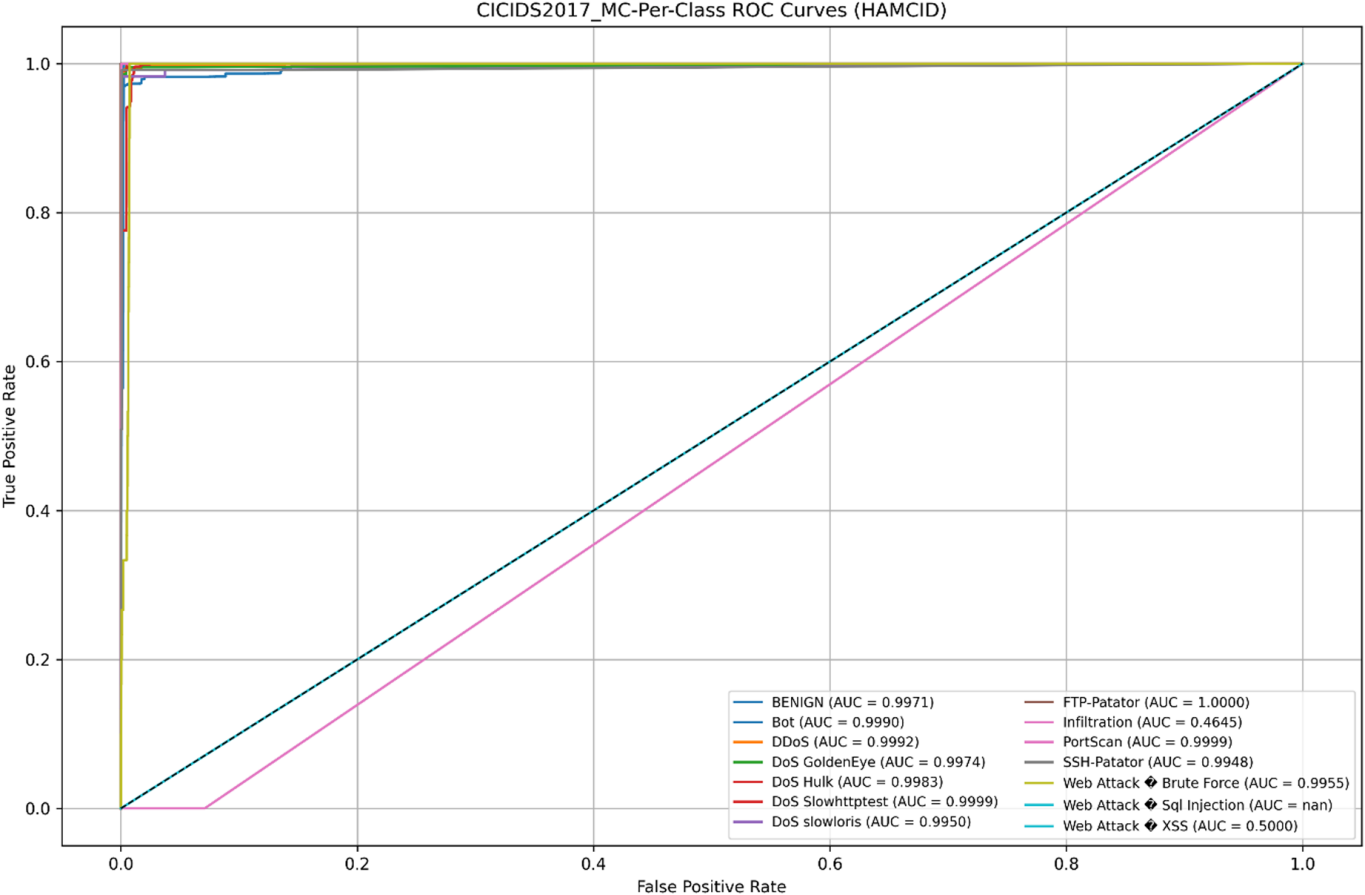
Per-Class ROC Curves _CICIDS2017 (Multiclass Classification).

Its learning dynamics and ROC analysis further support HAMC-ID’s superiority. Accuracy curves (Figs. 15 and 16) verify strong generalization across both datasets, whereas training and validation loss curves (Figs. 17 and 18) highlight smooth convergence with little overfitting. HAMC-ID’s highest AUC values, 0.9904 (UNSW-NB15) and 0.9978 (CICIDS2017), are highlighted in the micro-averaged ROC curves (Figs. 19 and 20). Strong performance even on minority classes, such Worms (0.9758 UNSW-NB15) and DoS Slowhttptest (0.8898 CICIDS2017), is further confirmed by per-class ROC analysis (Figs. 21 and 22).

**Fig. 23:**
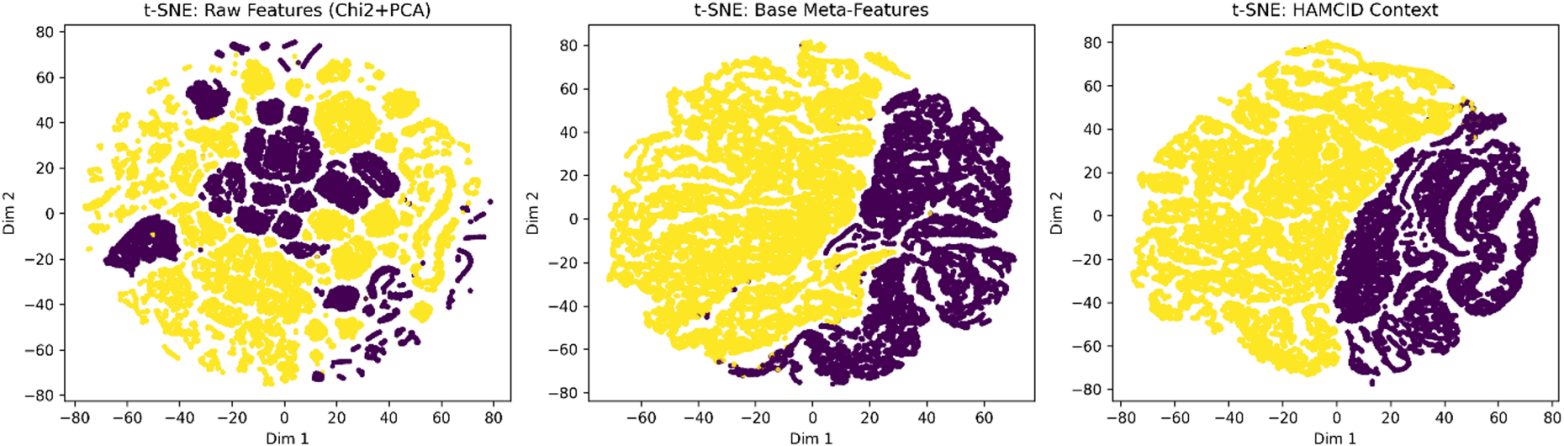
The t-SNE visualization of feature representations.

Lastly, Fig. 23 shows the discriminative power of HAMC-ID on both datasets using the t-SNE visualization of learnt feature representations. Different clusters are created between various attack types, including low-frequency classes, as well as between benign and malicious traffic. This image supports the quantitative findings by offering intuitive proof that the model successfully captures structural differences.

**Table 6:** Performance Metrics_UNSW-NB15(Multiclass Classification).

Classifier	Class	Precision	Recall	F1-Score	Accuracy (%)
Logistic regression	Analysis	0.1005	0.3159	0.1525	65.49
	Backdoor	0.0648	0.3884	0.1111	
	DoS	0.3485	0.3320	0.3401	
	Exploits	0.8029	0.4089	0.5418	
	Fuzzers	0.4430	0.6284	0.5197	
	Generic	0.9961	0.9729	0.9844	
	Normal	0.9857	0.7143	0.8283	
	Reconnaissance	0.2224	0.2252	0.2238	
	Shellcode	0.0817	0.7550	0.1474	
	Worms	0.0145	0.8000	0.0285	
Extra Trees	Analysis	0.1273	0.1925	0.1533	65.92
	Backdoor	0.0562	0.6245	0.1032	
	DoS	0.3577	0.2868	0.3183	
	Exploits	0.8317	0.4134	0.5522	
	Fuzzers	0.4180	0.6896	0.5205	
	Generic	0.9984	0.9727	0.9854	
	Normal	0.9998	0.7080	0.8290	
	Reconnaissance	0.7860	0.2573	0.3877	
	Shellcode	0.0768	0.8079	0.1402	
	Worms	0.0158	0.8000	0.0311	
XGBoost	Analysis	0.1167	0.4000	0.1807	76.9
	Backdoor	0.1005	0.3155	0.1524	
	DoS	0.3607	0.6038	0.4516	
	Exploits	0.8493	0.4475	0.5862	
	Fuzzers	0.5922	0.7830	0.6744	
	Generic	0.9992	0.9742	0.9865	
	Normal	0.9871	0.8302	0.9019	
	Reconnaissance	0.8258	0.8199	0.8228	
	Shellcode	0.2424	0.9272	0.3844	
	Worms	0.0463	0.8000	0.0875	
HAMC-ID	Analysis	0.3394	0.1757	0.2315	83.33
	Backdoor	0.1580	0.3455	0.2168	
	DoS	0.3604	0.7227	0.4810	
	Exploits	0.8250	0.5453	0.6566	
	Fuzzers	0.8427	0.7234	0.7785	
	Generic	0.9959	0.9809	0.9883	
	Normal	0.9627	0.9611	0.9619	
	Reconnaissance	0.8635	0.8024	0.8318	
	Shellcode	0.3570	0.8841	0.5086	
	Worms	0.1810	0.6000	0.2781	

**Table 7:** Performance Metrics_CICIDS2017(Multiclass Classification).

Classifier	Class	Precision	Recall	F1 Score	Accuracy (%)
Logistic Regression	BENIGN	0.9993	0.6141	0.7607	68.39
	Bot	0.0215	1	0.0421	
	DDoS	0.8616	0.9414	0.8998	
	DoS GoldenEye	0.2935	0.966	0.4502	
	DoS Hulk	0.4736	0.9698	0.6364	
	DoS Slowhttptest	0.1288	0.9636	0.2272	
	DoS slowloris	0.1381	0.8966	0.2394	
	FTP-Patator	0.0836	1	0.1543	
	Infiltration	0.0098	1	0.0194	
	PortScan	0.6912	0.9965	0.8162	
	SSH-Patator	0.0311	0.9915	0.0604	
	Web Attack – Brute Force	0.018	0.3	0.034	
	Web Attack – Sql Injection	0	0	0	
	Web Attack – XSS	0.009	0.8462	0.0178	
Extra Trees	BENIGN	0.9999	0.6823	0.8111	73.74
	Bot	0.0308	1	0.0597	
	DDoS	0.9135	0.9285	0.921	
	DoS GoldenEye	0.3368	0.9417	0.4962	
	DoS Hulk	0.4757	0.9607	0.6363	
	DoS Slowhttptest	0.6215	1	0.7666	
	DoS slowloris	1	0.8793	0.9358	
	FTP-Patator	0.1007	1	0.183	
	Infiltration	0	0	0	
	PortScan	0.674	0.9981	0.8047	
	SSH-Patator	0.0324	0.9915	0.0628	
	Web Attack – Brute Force	0.0561	0.2	0.0876	
	Web Attack – Sql Injection	0	0	0	
	Web Attack – XSS	0.05	1	0.0952	
XGBoost	BENIGN	0.9344	0.9717	0.9527	91.64
	Bot	0	0	0	
	DDoS	0.8947	0.591	0.7118	
	DoS GoldenEye	0	0	0	
	DoS Hulk	0.9536	0.6561	0.7773	
	DoS Slowhttptest	0.6786	0.1727	0.2754	
	DoS slowloris	0	0	0	
	FTP-Patator	0.9881	0.522	0.6831	
	Infiltration	0	0	0	
	PortScan	0.7027	0.9622	0.8122	
	SSH-Patator	0	0	0	
	Web Attack – Brute Force	0	0	0	
	Web Attack – Sql Injection	0	0	0	
	Web Attack – XSS	0	0	0	
HAMC-ID	BENIGN	0.9998	0.968	0.9836	97.26
	Bot	0.1071	1	0.1935	
	DDoS	0.9772	0.9879	0.9825	
	DoS GoldenEye	0.9393	0.9757	0.9571	
	DoS Hulk	0.888	0.9939	0.938	
	DoS Slowhttptest	0.8074	0.9909	0.8898	
	DoS slowloris	0.8976	0.9828	0.9383	
	FTP-Patator	0.9636	1	0.9815	
	Infiltration	0	0	0	
	PortScan	0.99	0.9994	0.9947	
	SSH-Patator	0.7091	0.9915	0.8269	
	Web Attack – Brute Force	0.0441	0.5	0.0811	
	Web Attack – Sql Injection	0	0	0	
	Web Attack – XSS	0.0593	0.5385	0.1069	

## Complexity analysis

The proposed HAMC-ID model introduces only modest computational overhead compared to traditional ensembles. GaussianNB, Logistic Regression, and XGBoost have training complexities of O(n⋅d), O(n⋅d⋅k) per iteration, and O (n⋅d⋅log n) per tree, respectively. The attention-based fusion module adds O(n⋅m^2^) overhead, which is negligible for small ensembles. In contrast, deep sequential models like BiLSTM require substantially more computation due to multiple stacked layers and large parameter spaces, resulting in longer training and inference times and higher hardware costs. By avoiding sequential deep architectures, HAMC-ID achieves high detection accuracy with low inference latency, making it suitable for real-time intrusion detection.

## Ablation study

The significance of each component to HAMC-ID is confirmed by the ablation study (Table 8). Restricting meta-features (probabilities only) or substituting attention with an MLP results in a minor decrease in accuracy and F1, highlighting the importance of the attention module in successful fusion. The biggest decline is caused by removing XGBoost, underscoring its significance as a base learner. While feature reduction to the top 50 features maintains competitive performance but falls short of the entire model, SMOTE and PCA have slight but consistently positive effects on efficiency and class balance. These findings collectively show that the most dependable and robust intrusion detection performance is obtained with the full HAMC-ID setup. It is important to note that the ablation study was conducted under controlled experimental settings (balanced subsets and fixed splits), so the absolute values differ from the reported binary classification accuracy (98.94%); the primary purpose here is to highlight the relative contribution of each component.

**Table 8:** Ablation study of HAMC-ID.

Variant	Accuracy	F1	Precision	Recall
HAMC-ID	0.9991	0.999	0.9993	0.999
MLP instead of Attention	0.9978	0.998	0.9981	0.998
Probs only	0.998	0.998	0.9985	0.998
Probs + Entropy	0.9985	0.999	0.9987	0.998
Without XGB	0.9915	0.992	0.9924	0.991
Without SMOTE	0.9987	0.999	0.9986	0.999
Without PCA	0.9989	0.999	0.999	0.999
Top-50 Features	0.9983	0.998	0.9986	0.998

## Threats to validity

In order to maintain openness, we recognize possible challenges to validity in four areas. Preprocessing decisions like feature selection, scaling, and oversampling can affect internal validity and may introduce hidden biases even when stratified splits and careful data management are used. Despite being widely used, benchmark datasets (UNSW-NB15, CICIDS2017) may not accurately reflect the complexity of real-world network traffic or zero-day attacks, which limits external validity. Binary classification experiments may mask issues in multi-class attack detection, and the use of standard classification metrics (accuracy, precision, recall, and F1-score) that do not evaluate factors like detection latency, computational overhead, or robustness against adversarial manipulation may compromise construct validity. The strength of inferences about superiority over baseline procedures may be impacted by the variation in conclusion validity caused by class imbalance, random initialization, and the lack of thorough statistical significance testing.

## Comparison with existing ensemble systems

A comparison of the suggested model with previous methods published by Thockchom et al.^3^ and Farhin Farha et al.^33^ is shown in Table 9. Precision and recall, two important performance variables, are compared across several assault categories. Thockchom et al.^3^ proposed a hybrid ensemble model by means of combination of classifiers such as Decision Tree (DT), Logistic Regression, Gaussian Naive Bayes, and Stochastic Gradient Descent (SGD). These techniques serve as a benchmark for comparison, demonstrating how well the suggested strategy works to identify a wider range of attacks with increased dependability.

These models showed lesser recall for Backdoor (8.41%) and Reconnaissance (7.98%), suggesting possible difficulties in accurately recognizing these attack types, while it showed great precision in detecting Analysis (98.80%) and Fuzzers (91.71%).

These models showed lesser recall for Backdoor (8.41%) and Reconnaissance (7.98%), suggesting possible difficulties in accurately recognizing these attack types, while it showed great precision in detecting Analysis (98.80%) and Fuzzers (91.71%).

A high level of precision was stated by Farhin Farha et al.^33^ for Normal (94.83%) and Generic (99.49%) traffic after with a mixture of K-Nearest Neighbors (KNN), Feedforward Neural Network (FNN), DT, and SGD classifiers. Nevertheless, the model’s performance significantly dropped for Worms traffic (6.86%). Yet, their model struggled to recognize Worms (6.86% precision) and Analysis (17.49% precision), suggesting that there are anomalies in the way they identify specific types of attacks. The suggested model, on the other hand, exhibits balanced performance across a variety of attack types by combining LR, ET, and XGBoost with an Attention Mechanism. In addition to maintaining high recall rates for Shellcode (88.41%), DoS (72.27%), and Normal (96.11%) traffic, it obtains the highest precision in Exploits (82.50%), Fuzzers (84.27%), Generic (99.59%), and Reconnaissance (86.35%). Remarkably, the suggested model addresses a serious flaw in the current methods by greatly increasing recall for Backdoor assaults (34.55%) when compared to other studies.

HAMC-ID demonstrates superior generalization and reliability, interpreting it more effective for real-world IDS involving varied threat vectors. whereas previous studies such as those by Thockchom et al.^3^ and Farhin Farha et al.^33^ attain commendable results in explicit attack types.

**Table 9:** Performance evaluation of the HAMC-ID with existing ensemble methods.

Author	Thockchom et al.^3^		Farhin Farha et al.^33^		Proposed model	
Classifier(s)	DT, LR, GNB + SGD		KNN, FNN, DT + SGD		LR, ET, XGB + Attention Meta	
Attack type	Precision	Recall	Precision Recall		Precision Recall	
Analysis	98.80	98.11	17.49	22.08	33.94	17.57
Backdoor	31.40	08.41	14.60	13.02	15.80	34.55
DOS	61.14	86.20	34.12	39.98	36.04	72.27
Exploits	60.14	61.30	68.14	65.06	82.50	54.53
Fuzzers	91.71	91.00	58.06	68.77	84.27	72.34
Generic	54.55	19.35	99.49	97.60	99.59	98.09
Normal	55.81	13.82	94.83	86.80	96.27	96.11
Reconnaissance	47.56	07.98	78.16	77.61	86.35	80.24
Shellcode	49.82	51.48	34.40	81.35	35.70	88.41
Worms	86.01	75.55	6.86	93.33	18.10	60.00

## Conclusion

The proposed HAMC-ID, a Hybrid Attention-Based Meta-Classifier that effectively combines XGBoost, Logistic Regression, and Extra Trees through a dynamic attention-based fusion mechanism. The model adaptively reweights base classifier outputs, enhancing detection in complex and imbalanced environments. The experimental valuation on the UNSW-NB15 dataset determines that the proposed HAMC-ID exceeds both individual classifiers and traditional ensemble techniques in terms of accuracy, precision, recall, and F1-score—particularly within the multiclass classification context. While maintaining high performance across all classes, its robust appropriateness to detect understated attack types, highlights its potential for everyday IDS applications. HAMC-ID attained an overall accuracy of 98.94%, dazzling its usefulness and generalizability. Furthermore, validation on both UNSW-NB15 and CICIDS2017 highlights HAMC-ID’s robustness and transferability, reinforcing its potential for real-world IDS deployments.

## Data Availability

UNSW-NB15 (https://research.unsw.edu.au/projects/unsw-nb15-dataset) CICIDS2017 (http://cicresearch.ca/CICDataset/CIC-IDS-2017/Dataset/CIC-IDS-2017).
